# Risk and Status of Gastrointestinal Cancer According to the International Standard Industrial Classification in Korean Workers

**DOI:** 10.3390/cancers14205164

**Published:** 2022-10-21

**Authors:** Soonsu Shin, Jun-Hyeok Choi, Kyung-Eun Lee, Jin-Ha Yoon, Wanhyung Lee

**Affiliations:** 1 Department of Preventive Medicine, Graduate School, Kyung Hee University, Seoul 02447, Korea; 2 Occupational Safety and Health Research Institute, Korea Occupational Safety and Health Agency, Ulsan 44429, Korea; 3 Department of Preventive Medicine, Yonsei University College of Medicine, Seoul 03722, Korea; 4 The Institute for Occupational Health, Yonsei University College of Medicine, Seoul 03722, Korea; 5 Department of Occupational and Environmental Medicine, Gil Medical Center, Gachon University College of Medicine, Incheon 21565, Korea

**Keywords:** cancer, gastrointestinal cancer, cancer incidence, standardized incidence ratios, occupation, the International Standard Industrial Classification of All Economic Activities

## Abstract

**Simple Summary:**

The risk of developing cancers varies depending on occupation, which is a well-known fact. It is crucial to investigate the risk of developing gastrointestinal (GI) cancer in the entire industry. In this study, we calculated the standardized incidence ratios (SIRs) of GI cancer by all industrial sectors as classified in the Korean Standard Industrial Classification (KSIC). There were noticeable differences among industries in the risk of GI cancer. The SIR of all GI cancer was highest among miners and quarriers, and second highest among transportation workers. These results suggest that further research is required to identify the risk factors present in each industry that contribute to the development of GI cancers. We believe this study can help to create tailored cancer prevention strategies for different industrial sectors.

**Abstract:**

To compare the risk of developing gastrointestinal (GI) cancer according to industrial groups, we performed a retrospective cohort study using the database of the Korea National Health Insurance Service (NHIS). We calculated the age-standardized incidence ratios (SIRs) and 95% confidence intervals (95% CIs) for the types of GI cancers according to the Korean Standard Industrial Classification (KSIC) compared with the whole employee population. The highest SIR for all GI cancer was found in the “Mining and quarrying” section (SIR, 1.30; 95% CI, 1.14–1.47), followed by the “Transportation and storage” section (SIR, 1.27; 95% CI, 1.24–1.30). Miners and quarriers had the highest risk of developing gastric cancer (SIR, 1.29; 95% CI, 1.06–1.55) and cancer of the liver and intrahepatic bile ducts (SIR, 1.48; 95% CI, 1.17–1.86). Transportation workers had the highest SIR of cancer of the lip, oral cavity, and pharynx (SIR, 1.27; 95% CI, 1.13–1.43) and cancers of the rectum, anus, and anal canal (SIR, 1.27; 95% CI, 1.19–1.35). There were distinct GI cancers with an elevated risk in each industry group. Our findings imply that distinct cancer prevention programs should be developed for each industrial sector.

## 1. Introduction

Estimating the incidence of cancer in relation to social structures is an important issue for cancer research. Occupation, one of the many factors that constitute the social structure, is recognized as a risk factor for cancer. Previous studies estimated that the total occupation-attributable fraction for all cancer varies at 2–8% [1]. Moreover, there are global differences in the standardized incidence ratios (SIRs) of various cancers according to occupation [2,3,4].

Gastrointestinal (GI) cancers account for approximately 4.8 million new cases each year and 26% of all cancer cases worldwide [5]. Several studies have shown that certain occupations increase the risk of developing GI cancer. Population-based case–control studies reported that farmers/gardeners, workers in hotel and restaurant industries, and workers exposed to dust had a considerably increased risk of developing squamous cell carcinoma of the esophagus [6,7]. Similarly, miners, workers in the leather industry, and those who were exposed to dust had a high risk of developing gastric cancer [8,9,10]. These studies investigated only the association between certain types of GI cancers and occupation, and there were limitations due to the small sample size.

Few studies have examined the incidence of GI cancer by type among industrial sectors using data from single-ethnic countries [2,4,11]. However, these studies have been limited because they did not use a detailed classification of industries and did not account for changes in occupations after cohort enrollment. Therefore, a systematic understanding of the relationship between the industry and GI cancer is still lacking. In view of these limitations, this study aimed to estimate SIRs of all types of GI cancer among industries using the Korean National Health Insurance Service (NHIS) database representing the entire Korean population.

## 2. Materials and Methods

### 2.1. Data and Study Participants

We used collaborated data from the Korean NHIS database and the Occupational Safety and Health Research Institute of Korea covering 2007 to 2015. All Korean citizens are required to have public health insurance which includes health examination, hospital visits, and medical care through the NHIS [12]. The NHIS covered approximately 98% of 52,034,424 individuals in the Republic of Korea in 2015 [13]. The NHIS data consisted of information on insurance qualifications and medical service use. Qualification data included basic information of subscribers such as age, sex, address, insurance status, occupation and industry category, and income level. We selected the working population based on the type of insurance from the qualification data. The medical use data comprised details of hospital visits, diagnoses, and treatments. According to the standard guidelines of the Korean Standard Classification of Diseases and Causes of Death, Fourth Edition, which matched to the International Classification of Diseases, Tenth Revision (ICD-10); all recorded diseases were classified [14].

Occupation-based epidemiologic studies usually had limitations because it was hard to track changes in their job or employment status during the follow-up period. To overcome these problems, previous studies have attempted various methods to evaluate working status [15,16]. The NHIS annually established qualification data for the job and industry categories on the last day of the year. We defined workers with their occupation as an initially registered qualification in 2007, and subjects who changed their working status from non-worker to worker after commencing follow-up were enrolled in the study.

### 2.2. Cancers

Using the ICD-10 classification “C00-C97 Malignant neoplasms,” cancers were identified as inpatients with claims information. Malignant neoplasms were categorized into seven groups and 27 subgroups according to the Korean Standard Classification of Diseases from the ICD-10 code depending on where cancer occurs in the human organs [17]. We used the following code of GI cancers: malignant neoplasms of the lip, oral cavity, and pharynx (C00–C14); esophagus (C15); stomach (C16); colon (C18); rectosigmoid junction, rectum, anus, and anal canal (C19–C21); liver and intrahepatic bile ducts (C22); pancreas (C25); and other digestive organs (C17, C23, C24, and C26) [18].

### 2.3. Definition of Industrial Classification

The Korean Standard Industrial Classification (KSIC) was developed by the Korea National Statistical Office following the 4th revision of the International Standard Industrial Classification of All Economic Activities (ISIC) in 2008 [19]. The ISIC categorized industry using the top-down method as follows: Section–Division–Group–Class. The detailed classification level can be converted according to the national scheme. The KSIC had 21 sections: A. agriculture, forestry and fishing; B. mining and quarrying; C. manufacturing; D. electricity, gas, steam, and air conditioning supply; E. water supply, sewerage, waste management, and remediation; F. construction; G. wholesale and retail trade and repair of motor vehicles and motorcycles; H. transportation and storage; I. accommodation and food service activities; J. information and communication; K. financial and insurance activities; L. real estate activities; M. professional, scientific, and technical activities; N. administrative and support service activities; O. public administration and defence and compulsory social security; P. education; Q. human health and social work activities; R. arts, entertainment, and recreation; S. other service activities; T. activities of households as employers and undifferentiated goods- and services-producing activities of households for own use; U. activities of extraterritorial organizations and bodies. In this study, there was unclassified industry information in the NHIS, and the “not confirmed” class was added.

### 2.4. Statistical Analysis

Cancer cases and status according to sex, age group, and cancer site were calculated. The age SIRs and 95% confidence intervals (CIs) of all and each GI cancer were estimated according to the KSIC. The KSIC had multiple sections and sub-sectional division levels. Gastrointestinal cancer also had various sub-types according to the organ. Thus, we conducted analysis with multiple stratifications of cancer site and level of the KSIC. The SIRs were calculated as the weighted average of age-specific incidence density rates. We stratified age and sex in 5-year groups and set the entire working population in the current data as the reference group. We used the indirect standardization method, which uses age-specific gastrointestinal cancer incidence rates and the number of person-years in each age group of the entire working population in the current data to calculate the expected number of gastrointestinal cancer cases after adjusting for age. The ratio of the observed to the expected number of cases was the SIRs. The 95% CIs were calculated according to the distribution of the Poisson. In this study, if the SIRs were more than 1 and the lower limit of the 95% CI was also more than 1 (or the SIRs was less than 1 and the upper limit of the 95% CI was also less than 1), the risk of gastrointestinal cancer was considered to be significantly higher (or lower) among the workers in each of the KSIC than in the entire working population. The SAS version 9.4 (SAS Institute, Cary, NC, USA) was used for all analyses.

## 3. Results

During the follow-up period, 92,137 (0.83%) GI cancer cases were observed in 11,050,398 participants (Table 1). There were 77,493 cases of GI cancer in male workers and 14,644 cases of GI cancer in female workers. The older population showed an increased prevalence of GI cancer.

Table 2 shows the sex and type of cancer-specific cases. According to cancer type, “Malignant neoplasm of stomach” had the highest cases (33,524), and “Malignant neoplasm of esophagus” had the lowest cases (1864) in both sexes. In male workers, “Malignant neoplasms of the liver and intrahepatic bile ducts” showed secondary high cases (17,009) and, in female workers, “Malignant neoplasms of the colon” showed secondary high cases (3073).

Table 3 presents the SIRs and 95% CI of all GI cancers according to the section of the KSIC with sex stratification. Before gender stratification, the highest significantly increased risk was found in the “Mining and quarrying” section (SIR, 1.30; 95% CI, 1.14–1.47), followed by the “Transportation and storage” section (SIR, 1.27; 95% CI, 1.24–1.30).

**Figure 1:**
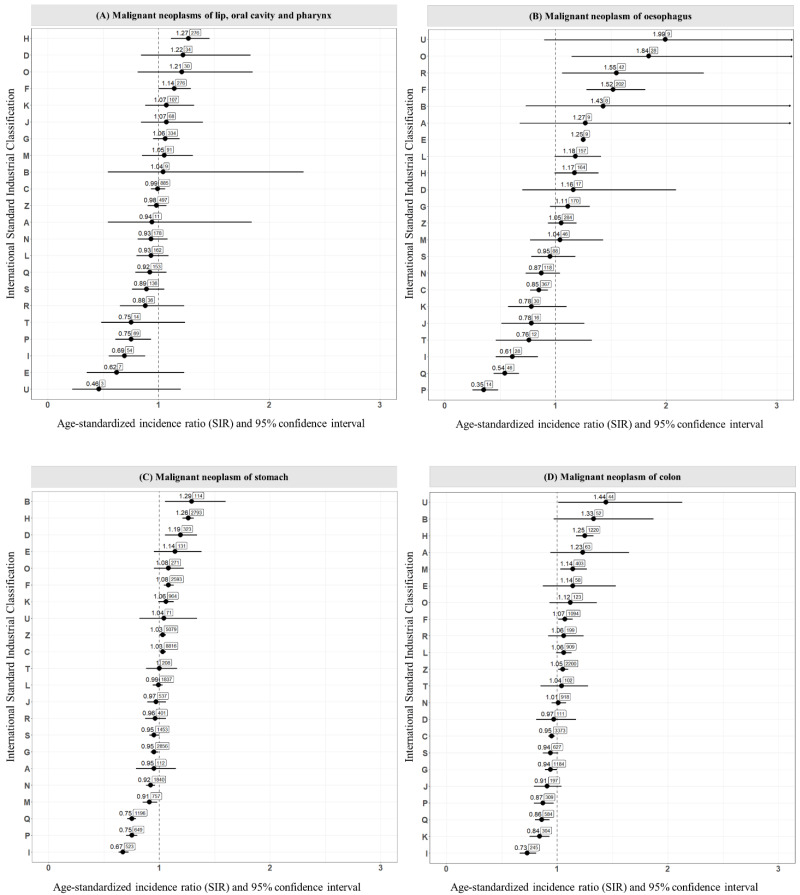

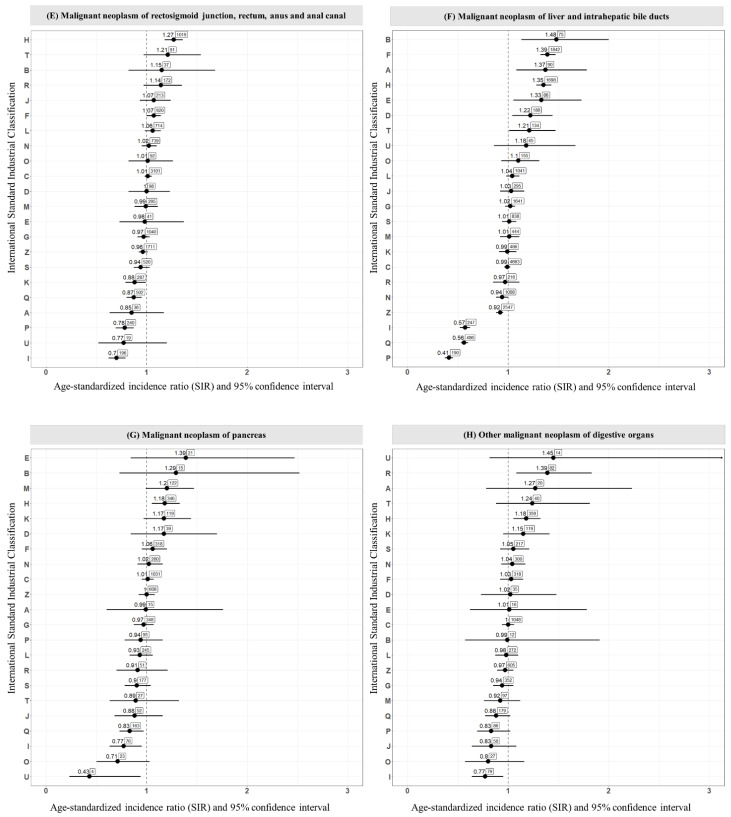
Risk and cases of each gastrointestinal cancer site according to the Korean National Standardized Industrial Classification. Footnote: A. agriculture, forestry and fishing, B. mining and quarrying, C. manufacturing, D. electricity, gas, steam and air conditioning supply, E. water supply; sewerage, waste management and remediation, F. construction, G. wholesale and retail trade; repair of motor vehicles and motorcycles, H. transportation and storage, I. accommodation and food service activities, J. information and communication, K. financial and insurance activities, L. real estate activities, M. professional, scientific and technical activities, N. administrative and support service activities, O. public administration and defence; compulsory social security, P. education, Q. human health and social work activities, R. arts, entertainment and recreation, S. other service activities, T. activities of households as employers; undifferentiated goods- and services-producing activities of households for own use, U. activities of extraterritorial organizations and bodies, Z. not confirmed. Number above dot = SIR, number in box = cases.

The risk and number of cases of each GI cancer site according to the KSIC are described in Figure 1. The highest risk of specific cancer by section of the KSIC was as follows: “Malignant neoplasms of lip, oral cavity and pharynx” for -“H. Transportation”; “Malignant neoplasm of esophagus” for “U. Activities of extraterritorial organizations and bodies”; “Malignant neoplasm of stomach” for “B. Mining and quarrying”; “Malignant neoplasm of colon” for -“U. Activities of extraterritorial organizations and bodies”; “Malignant neoplasm of rectosigmoid junction, rectum, anus, and anal canal” for -“H. Transportation”; “Malignant neoplasm of liver and intrahepatic bile ducts” for “B. Mining and quarrying”; “Malignant neoplasm of pancreas” for “E. Water supply; sewerage, waste management and remediation”; and “Other malignant neoplasm of digestive organs” for “U. Activities of extraterritorial organizations and bodies”.

Detailed expected and observed cases with SIRs (95% CI) according to the division of the KSIC and type of cancer are presented in the Supplementary Tables.

## 4. Discussion

The current study aimed to compare the risk of GI cancers among various industry groups in Korea with that of the working population as a whole. Significant differences were observed in the SIRs of GI cancers between industries. This outcome is assumed to be the result of exposure to different hazardous factors in each industrial category.

According to an analysis of the SIRs of all GI cancers by industrial groups, the “Mining and quarrying” section had the highest SIR of all GI cancers. Moreover, workers in mining and quarrying industries had the highest risk of developing gastric cancer and liver and intrahepatic bile duct cancer. Previous studies have reported that miners and quarriers have a higher risk of incidence and mortality of gastric cancer than the general population [2,9,10,20]. Workers exposed to inorganic dust (mainly silica dust) had a higher risk of developing liver cancer [21]. However, the SIR of liver cancer in miners and quarriers was not statistically significant in a retrospective cohort study of 15 million participants in five Nordic countries [2].

External airborne agents (EAA), such as mineral dust, vapor, and engine exhaust, are substances that are released into the atmosphere and exposed to workers [18]. Miners and quarriers are exposed to EAA in their working environments [22,23]. EAA is known to cause lung cancer and various respiratory system diseases, including pneumoconiosis [24,25]. Several studies have demonstrated that workers exposed to EAA have an increased risk of esophageal cancer, stomach cancer, and all GI cancers [18,26,27,28,29,30]. A previous study postulated that the lung clearance system can release inhaled EAA into the oral cavity, where they are constantly swallowed and enter the digestive tract [31]. EAA causes inflammation of the digestive tract [32] and acts as a mutagen in tumor-suppressor genes or proto-oncogenes [33,34]. Additionally, mines and quarries have lately been identified as major emitters of particulate matter of ≤2.5 μm diameter (PM2.5), and it is known that workers in these locations are exposed [35,36]. In a review of the literature, no data were found on the association between occupational exposure to PM2.5 and liver cancer. However, some studies have reported that the incidence of liver cancer increases with increasing environmental exposure to PM2.5 [37,38].

The SIR of esophageal cancer and the SIR of colon cancer were the highest in extraterritorial organization employees. However, the SIR of esophageal cancer in extraterritorial organization employees was not statistically significant. The industry group with the highest statistically significant risk of esophageal cancer was “public administration and defence; compulsory social security”. These outcomes are attributable to their relatively high socioeconomic status and level of education, which enable the early diagnosis of esophageal and colon cancer by endoscopy. Under the current healthcare system in Korea, individuals with low socioeconomic status and educational levels have limited access to hospitals and frequently unmet their needs for healthcare services [39,40,41]. Previous studies have shown that low socioeconomic levels increase the risk and mortality of esophageal cancer in early stage [42,43].

Compared to the whole working population, workers in the transportation industry had the highest risk of cancer of the lip, oral cavity, pharynx, rectum, and anus and the second highest risk of all GI cancers. The results of the present study are consistent with findings from earlier studies. In Nordic nations, there is an increase in the SIR of lip, pharyngeal, and rectal cancers among drivers and transportation workers [2,44].

Transportation workers are exposed to hazardous health factors, such as vehicle exhaust, night shift work, and physical factors (e.g., sunlight and vibration) in the working environment [45,46]. The International Agency for Research on Cancer (IARC) identified diesel engine exhaust as “Group 1, carcinogenic to humans” and gasoline engine exhaust as “Group 2B, possibly carcinogenic to humans” [47]. One case–control study revealed that engine exhaust increases the risk of lip cancer [48]. Furthermore, rectal cancer risks were also elevated after prolonged exposure to high amounts of vehicle exhaust, but the association between diesel exhaust and colon cancer was not significant [49,50]. Drivers are exposed to sunlight coming through the window. Sun exposure is one of the risk factors for lip cancer, and occupational exposure to sunlight increases the risk of lip cancer [51]. IARC designated night shift work as “Group 2A, probably carcinogenic to humans” [52], and a meta-analysis revealed that night-shift work increased the risk of GI cancer regardless of sex [53]. As transportation workers are exposed to various hazardous health factors that increase the risk of cancer, the transportation industry is one of the most vulnerable to GI cancer.

Another important finding was the highest SIR for pancreatic cancer in the water supply, sewage, waste management, and remediation sectors. However, the difference was not statistically significant. Despite numerous studies on the occupational health risks of sanitation employees, few have focused on the development of cancer in sanitation workers. Several researchers have attempted to study the risk of cancer in sanitation workers, but the results have been inconsistent [54].

Sanitation workers are exposed to various chemical and biological factors in the workplace, such as polycyclic aromatic hydrocarbons (PAH), volatile organic compounds, hydrogen sulfide, and endotoxins [55,56]. Based on meta-analyses, occupational exposure to PAH has been associated with an increased risk of pancreatic cancer [57]. Endotoxins, lipopolysaccharides found in the cell membrane of Gram-negative bacteria, have recently been identified as a trigger of pancreatitis [58,59]. Chronic pancreatitis may lead to pancreatic cancer [60]. Nevertheless, there is a lack of evidence on whether endotoxin is a direct risk factor for pancreatic cancer. Further studies on the effects of endotoxins on pancreatic cancer are required.

This study is valuable in that it is the first to examine the risk of GI cancer across an entire industry in a country considering changes in the occupation of participants. Our findings encourage policymakers, regulators, and epidemiologists to focus on the health of specific industries. However, this study has several limitations. We did not calculate the hazard ratio of cancer after adjusting for covariates such as regional disparity, alcohol consumption, and smoking. Therefore, our results should only be interpreted as requiring attention to specific occupations because they had a high incidence of certain GI cancers. Additionally, our findings cannot be extrapolated to all individuals, because this was an ecological study. As this study is based on industrial classification rather than exposure assessment, it limits the identification of risk factors associated with increased risk of GI cancer. We could not consider the hazardous health factors that workers are actually exposed to in their workplace. In the future, we plan to analyze the risk of cancer by considering individual factors after resolving the issue of personal information. Furthermore, instead of using a histological diagnosis, we defined cancer by using the ICD-10 code in the NHS database. However, according to previous studies that define cancer using the ICD-10 code from the NHIS database, medical records containing cancer diagnoses are regarded as reliable [61].

## 5. Conclusions

In this study, we attempted to understand the relationship between industrial structure and the risk of GI cancer. This large retrospective cohort study revealed that workers in the mining and quarrying sectors had the highest risk of incidence of all GI cancers, gastric cancer, and cancers of the liver and intrahepatic bile ducts. Transportation workers had the second highest risk of all GI cancers and had the highest risk of lip, oral cavity, pharynx, rectum, and anus cancers. The risk of pancreatic cancer was the highest in the “water supply, sewage, waste management, and remediation” industry. Different health risks in each industry are believed to be the reason for the differences in the incidence of GI cancer by industry group. Further research is needed to address the various occupational risks of GI cancer in each industry. In our opinion, our findings can contribute to the establishment of customized cancer prevention plans for various industrial sectors.

## Figures and Tables

**Table 1:** Descriptive characteristics of study participants and gastrointestinal cancer cases among workers during the follow-up period.

	Total Participants	Gastrointestinal Cancer Cases (%)
Total	11,050,398	92,137 (0.83)
Sex		
Male	7,362,615	77,493 (1.05)
Female	3,687,783	14,644 (0.38)
Age		
25–29	1,853,391	5006 (0.3)
30–34	1,907,448	10,913 (0.6)
35–39	1,925,593	22,531 (1.2)
40–44	1,697,347	36,268 (2.1)
45–49	1,494,043	59,881 (4.0)
50–54	1,147,382	77,070 (6.7)
55–59	657,070	66,642 (10.1)
60–64	368,124	50,779 (13.8)

**Table 2:** Sex-specific gastrointestinal cancer status according to the organ during the follow-up period.

Type of Cancer	ICD-10	Cases (%)		
		Total Workers	Male Workers	Female Workers
All gastrointestinal cancer	C00–C26	92,137	77,493	14,644
Malignant neoplasms of lip, oral cavity and pharynx	C00–C14	3430 (3.72)	2907 (3.75)	523 (3.57)
Malignant neoplasm of esophagus	C15	1864 (2.02)	1800 (2.32)	64 (0.44)
Malignant neoplasm of stomach	C16	33,524 (36.38)	27,840 (35.93)	5684 (38.81)
Malignant neoplasm of colon	C18	14,319 (15.54)	11,246 (14.51)	3073 (20.98)
Malignant neoplasm of rectosigmoid junction, rectum, anus and anal canal	C19–C21	12,083 (13.11)	9872 (12.74)	2211 (15.10)
Malignant neoplasm of liver and intrahepatic bile ducts	C22	18,415 (19.99)	17,009 (21.95)	1406 (9.60)
Malignant neoplasm of pancreas	C25	4175 (4.53)	3414 (4.41)	761 (5.20)
Other malignant neoplasm of digestive organs	C17, C23–C24, C26	4327 (4.70)	3405 (4.39)	922 (6.30)

**Table 3:** Age-standardized incidence ratios (SIRs) and 95% confidence intervals (CI) of all gastrointestinal cancer according to sections of the Korean National Standardized Industrial Classification.

Korean National Standardized Industrial Classification			Total Workers		Male Workers		Female Workers	
Section	Divisions	Description	Cases	SIR (95% CI)	Cases	SIR (95% CI)	Cases	SIR (95% CI)
A	01–03	Agriculture, forestry and fishing	356	1.09 (0.98–1.22)	313	1.09 (0.97–1.23)	43	0.96 (0.72–1.32)
B	05–08	Mining and quarrying	322	1.30 (1.14–1.47)	298	1.15 (1.02–1.30)	24	1.51 (0.93–2.66)
C	10–33	Manufacturing	23,284	1.00 (0.99–1.01)	20,187	0.98 (0.96–0.99)	3097	1.00 (0.97–1.04)
D	35	Electricity, gas, steam and air conditioning supply	845	1.13 (1.05–1.21)	819	0.99 (0.93–1.07)	26	0.82 (0.58–1.20)
E	36–38	Water supply; sewerage, waste management and remediation	369	1.15 (1.03–1.28)	342	1.10 (0.98–1.23)	27	0.89 (0.63–1.32)
F	41–42	Construction	7563	1.15 (1.12–1.18)	7072	1.03 (1.01–1.05)	491	1.13 (1.03–1.25)
G	45–47	Wholesale and retail trade; repair of motor vehicles and motorcycles	7925	0.97 (0.95–0.99)	6536	0.99 (0.96–1.01)	1389	0.98 (0.93–1.03)
H	49–52	Transportation and storage	7875	1.27 (1.24–1.30)	7674	1.11 (1.08–1.13)	201	0.92 (0.80–1.05)
I	55–56	Accommodation and food service activities	1448	0.67 (0.64–0.70)	741	0.91 (0.85–0.98)	707	0.95 (0.88–1.02)
J	59–63	Information and communication	1428	0.98 (0.93–1.03)	1266	0.90 (0.86–0.95)	162	0.96 (0.82–1.12)
K	64–66	Financial and insurance activities	2416	0.99 (0.96–1.03)	2038	0.90 (0.87–0.94)	378	1.06 (0.95–1.18)
L	68	Real estate activities	5337	1.02 (0.99–1.04)	4590	1.03 (1.00–1.06)	747	1.00 (0.93–1.08)
M	70–73	Professional, scientific and technical activities	2255	1.00 (0.96–1.04)	1987	0.95 (0.91–1.00)	268	0.88 (0.78–0.98)
N	74–76	Administrative and support service activities	5381	0.96 (0.94–0.99)	4170	1.06 (1.03–1.10)	1211	1.05 (0.99–1.11)
O	84	Public administration and defence; compulsory social security	749	1.07 (0.99–1.15)	646	1.09 (1.01–1.19)	103	0.93 (0.77–1.12)
P	85	Education	1652	0.71 (0.68–0.74)	686	0.91 (0.85–0.98)	966	0.97 (0.91–1.03)
Q	86–87	Human health and social work activities	3309	0.76 (0.74–0.78)	1765	0.94 (0.90–0.99)	1544	0.99 (0.94–1.04)
R	90–91	Arts, entertainment and recreation	1199	1.03 (0.98–1.10)	951	1.08 (1.01–1.15)	248	1.16 (1.01–1.33)
S	94–96	Other service activities	4056	0.96 (0.93–0.99)	3307	1.01 (0.98–1.05)	749	0.97 (0.90–1.04)
T	98	Activities of households as employers; undifferentiated goods- and services-producing activities of households for own use	628	1.07 (0.98–1.16)	539	1.06 (0.97–1.16)	89	1.10 (0.89–1.39)
U	99	Activities of extraterritorial organizations and bodies	209	1.09 (0.95–1.27)	187	1.07 (0.92–1.25)	22	0.98 (0.65–1.56)
Not confirmed			13,531	1.00 (0.98–1.01)	11,379	0.97 (0.95–0.99)	2152	1.01 (0.97–1.06)

## Data Availability

The data are not publicly available due to the data belonging to the Korean government.

## Supplementary Materials

The following supporting information can be downloaded at: https://www.mdpi.com/article/10.3390/cancers14205164/s1, Table S1. Age-standardized incidence ratios (SIR) and 95% confidence interval (CI) of all gastrointestinal cancer according to divisions of the Korean Standard Industrial Classification (KSIC). Table S2. Age-standardized incidence ratios (SIR) and 95% confidence interval (CI) of malignant neoplasms of lip, oral cavity and pharynx according to divisions of the Korean Standard Industrial Classification (KSIC). Table S3. Age-standardized incidence ratios (SIR) and 95% confidence interval (CI) of malignant neoplasm of oesophagus according to divisions of the Korean Standard Industrial Classification Table S4. Age-standardized incidence ratios (SIR) and 95% confidence interval (CI) of malignant neoplasm of stomach according to divisions of the Korean Standard Industrial Classification (KSIC). Table S5. Age-standardized incidence ratio (SIR) and 95% confidence interval (CI) of malignant neoplasm of colon according to divisions of the Korean Standard Industrial Classification (KSIC). Table S6. Age-standardized incidence ratio (SIR) and 95% confidence interval (CI) of malignant neoplasm of rectosigmoid junction, rectum, anus and anal canal according to divisions of the Korean Standard Industrial Classification (KSIC). Table S7. Age-standardized incidence ratios (SIR) and 95% confidence interval (CI) of malignant neoplasm of liver and intrahepatic bile ducts according to divisions of the Korean Standard Industrial Classification (KSIC). Table S8. Age-standardized incidence ratios (SIR) and 95% confidence interval (CI) of malignant neoplasm of pancreas according to divisions of the Korean Standard Industrial Classification (KSIC). Table S9. Age-standardized incidence ratios (SIR) and 95% confidence interval (CI) of other malignant neoplasm of digestive organs according to divisions of the Koreandigestf Standard Industrial Classification (KSIC).
