# Supplementary material for: Risk and Status of Gastrointestinal Cancer According to the International Standard Industrial Classification in Korean Workers
Source: Cancers (Basel). 2022 Oct 21;14(20):5164. doi: 10.3390/cancers14205164 (PMC9600415; doi:10.3390/cancers14205164)
Supplement: Supplementary file 1 [file cancers-14-05164-s001.zip › cancers-1930917-supplementary.pdf]

Supplementary Table S1. Age-standardized incidence ratio (SIR) and 95% confidence interval (CI) of all gastrointestinal tract cancer according to divisions of the Korean Standard Industrial Classification (KSIC).

KSIC		Total workers			Male workers			Female workers		
Divisions	Description	Expected cases	Observed cases	SIR (95% CI)	Expected cases	Observed cases	SIR (95% CI)	Expected cases	Observed cases	SIR (95% CI)
01	Agriculture	172.25	158	0.92 (0.79-1.07)	145.41	134	0.92 (0.77-1.09)	25.26	24	1.41 (0.61-1.41)
02	Forestry	40.51	62	1.53 (1.13-2.14)	41.64	59	1.42 (1.08-1.83)	2.95	3	1.02 (0.21-2.97)
03	Fishing and aquaculture	113.83	136	1.19 (0.99-1.45)	99.44	120	1.21 (1.00-1.44)	16.56	16	0.97 (0.55-1.57)
05	Mining of coal, crude petroleum and natural gas	162.14	221	1.36 (1.17-1.60)	169.54	203	1.20 (1.04-1.37)	10.39	18	1.73 (1.03-2.74)
06	Mining of metal ores	14.27	16	1.12 (0.67-2.04)	14.72	16	1.09 (0.62-1.77)	0.97	0	0.00 (0.00-3.79)
07	Mining of non-metallic minerals, except fuel	56.22	70	1.25 (0.96-1.65)	59.82	66	1.10 (0.85-1.40)	3.08	4	1.30 (0.35-3.33)
08	Mining support service activities	15.70	15	0.96 (0.59-1.68)	15.44	13	0.84 (0.45-1.44)	1.41	2	1.42 (0.17-5.12)
10	Manufacture of food products	1,208.52	1,044	0.86 (0.82-0.91)	697.45	710	1.02 (0.94-1.10)	317.77	334	1.05 (0.94-1.17)
11	Manufacture of beverages	51.49	46	0.89 (0.68-1.20)	46.33	43	0.93 (0.67-1.25)	6.39	3	0.47 (0.10-1.37)
12	Manufacture of tobacco products	3.78	3	0.79 (0.30-3.06)	3.43	2	0.58 (0.07-2.11)	0.45	1	2.24 (0.06-12.48)
13	Manufacture of textiles, except apparel	1,095.48	979	0.89 (0.84-0.95)	801.73	748	0.93 (0.93-0.87)	222.26	231	1.04 (0.91-1.18)
14	Manufacture of wearing apparel, clothing accessories and fur articles	296.02	212	0.72 (0.64-0.81)	158.89	139	0.87 (0.74-1.03)	86.74	73	0.84 (0.66-1.06)
15	Manufacture of leather, luggage and footwear	202.10	197	0.97 (0.85-1.12)	144.53	149	1.03 (0.87-1.21)	41.82	48	1.15 (0.85-1.52)
16	Manufacture of wood and of products of wood and cork; except furniture	306.67	348	1.13 (1.01-1.27)	288.42	317	1.10 (0.98-1.23)	32.93	31	0.94 (0.64-1.34)
17	Manufacture of pulp, paper and paper products	330.67	401	1.21 (1.09-1.35)	312.96	355	1.13 (1.02-1.26)	34.78	46	1.21 (1.10-1.34)
18	Printing and reproduction of recorded media	702.22	661	0.94 (0.87-1.01)	597.32	548	0.92 (0.84-1.00)	115.81	113	0.98 (0.80-1.17)
19	Manufacture of coke, briquettes and refined petroleum products	83.17	95	1.14 (0.92-1.43)	92.80	92	0.99 (0.80-1.22)	2.66	3	1.13 (0.23-3.29)
20	Manufacture of chemicals and chemical products; except pharmaceuticals and medicinal chemicals	1,138.21	1,141	1.00 (0.95-1.06)	1,070.76	1,002	0.94 (0.88-1.00)	125.27	139	1.11 (0.93-1.31)
21	Manufacture of pharmaceuticals, medicinal chemical and botanical products	80.71	65	0.81 (0.65-1.01)	66.58	50	0.75 (0.56-0.99)	13.65	15	1.10 (0.62-1.81)
22	Manufacture of rubber and plastics products	433.38	445	1.03 (0.93-1.13)	374.08	372	0.99 (0.90-1.10)	61.97	73	1.18 (0.92-1.48)
23	Manufacture of other non-metallic mineral products	496.77	532	1.07 (0.98-1.17)	488.16	479	0.98 (0.90-1.07)	44.12	53	1.20 (0.90-1.57)
24	Manufacture of basic metals	1,151.51	1,331	1.16 (1.09-1.23)	1,201.55	1,253	1.04 (0.99-1.10)	72.57	78	1.07 (0.85-1.34)
25	Manufacture of fabricated metal products, except machinery and furniture	1,061.23	1,148	1.08 (1.02-1.15)	1,024.27	1,050	1.03 (0.96-1.09)	103.64	98	0.95 (0.77-1.15)
26	Manufacture of electronic components, computer; visual, sounding and communication equipment	1,433.00	1,258	0.88 (0.83-0.93)	1,107.68	981	0.89 (0.83-0.94)	283.42	277	0.98 (0.87-1.10)

27	Manufacture of medical, precision and optical instruments, watches and clocks	152.41	147	0.96 (0.82-1.14)	123.63	117	0.95 (0.78-1.13)	26.60	30	1.13 (0.76-1.61)
28	Manufacture of electrical equipment	646.57	643	0.99 (0.92-1.08)	581.72	565	0.96 (0.88-1.04)	81.54	78	0.96 (0.76-1.19)
29	Manufacture of other machinery and equipment	1,440.82	1,524	1.06 (1.00-1.11)	1,416.29	1,403	0.99 (0.94-1.04)	131.28	121	0.92 (0.76-1.10)
30	Manufacture of motor vehicles, trailers and semitrailers	2,148.71	2,068	0.96 (0.92-1.00)	2,036.74	1,840	0.90 (0.86-0.95)	222.15	228	1.03 (0.90-1.17)
31	Manufacture of other transport equipment	1,154.34	1,297	1.12 (1.06-1.19)	1,238.86	1,233	1.00 (0.94-1.05)	57.72	64	1.11 (0.85-1.42)
32	Manufacture of furniture	219.32	210	0.96 (0.84-1.10)	196.62	185	0.94 (0.81-1.09)	27.81	25	0.90 (0.58-1.33)
33	Other manufacturing	7,414.74	7,489	1.01 (0.99-1.03)	6,612.11	6,554	0.99 (0.97-1.02)	975.73	935	0.96 (0.90-1.02)
35	Electricity, gas, steam and air conditioning supply	749.40	845	1.13 (1.05-1.21)	822.45	819	0.99 (0.93-1.07)	31.81	26	0.82 (0.53-1.20)
36	Water supply	49.86	47	0.94 (0.71-1.27)	54.06	45	0.83 (0.61-1.11)	2.49	2	0.80 (0.10-2.90)
37	Sewage, wastewater, human and animal waste treatment services	41.55	42	1.01 (0.75-1.41)	36.19	38	1.05 (0.74-1.44)	5.45	4	0.73 (0.20-1.88)
38	Waste collection, treatment and disposal activities; materials recovery	230.23	280	1.22 (1.07-1.39)	221.12	259	1.17 (1.03-1.32)	22.28	21	0.94 (0.58-1.44)
41	General construction	4,085.77	4,584	1.12 (1.09-1.16)	4,271.98	4,295	1.01 (0.98-1.04)	259.81	289	1.11 (0.99-1.25)
42	Specialized construction activities	2,501.23	2,979	1.19 (1.15-1.24)	2,593.38	2,777	1.07 (1.03-1.11)	172.80	202	1.17 (1.01-1.34)
45	Sale of motor vehicles and parts	306.82	361	1.18 (1.05-1.32)	301.16	324	1.08 (0.96-1.20)	28.44	37	1.30 (0.92-1.79)
46	Wholesale trade on own account or on a fee or contract basis	3,928.74	3,807	0.97 (0.94-1.00)	3,273.32	3,202	0.98 (0.94-1.01)	645.65	605	0.94 (0.86-1.01)
47	Retail trade, except motor vehicles and motorcycles	3,905.78	3,757	0.96 (0.93-0.99)	3,039.89	3,010	0.99 (0.96-1.03)	750.49	747	1.00 (0.93-1.07)
49	Land transport and transport via pipelines	1,193.50	1,468	1.23 (1.16-1.30)	1,310.29	1,412	1.08 (1.02-1.14)	50.38	56	1.11 (0.84-1.44)
50	Water transport	352.05	444	1.26 (1.14-1.40)	398.25	439	1.10 (1.00-1.21)	10.05	5	0.50 (0.16-1.16)
51	Air transport	42.80	56	1.31 (0.97-1.81)	44.49	52	1.17 (0.87-1.53)	3.23	4	1.24 (0.34-3.17)
52	Warehousing and support activities for transportation	4,630.21	5,907	1.28 (1.24-1.31)	5,182.83	5,771	1.11 (1.08-1.14)	155.72	136	0.87 (0.73-1.03)
55	Accommodation	446.11	370	0.83 (0.76-0.91)	256.68	249	0.97 (0.85-1.10)	116.30	121	1.04 (0.86-1.24)
56	Food and beverage service activities	1,707.20	1,078	0.63 (0.60-0.66)	554.83	492	0.89 (0.81-0.97)	629.24	586	0.93 (0.86-1.01)
59	Motion picture, video and television program production, sound recording and music publishing activities	55.38	44	0.79 (0.61-1.05)	52.46	39	0.74 (0.53-1.02)	6.87	5	0.73 (0.24-1.70)
60	Broadcasting activities	41.48	42	1.01 (0.75-1.41)	26.29	22	0.84 (0.52-1.27)	12.12	20	1.65 (1.01-2.55)
61	Postal activities and telecommunications	555.79	554	1.00 (0.92-1.09)	578.07	513	0.89 (0.81-0.97)	40.70	41	1.01 (0.72-1.37)
62	Computer programming, consultancy and related activities	322.52	314	0.97 (0.87-1.09)	301.87	273	0.90 (0.80-1.02)	42.23	41	0.97 (0.70-1.32)
63	Information service activities	483.77	474	0.98 (0.90-1.07)	443.80	419	0.94 (0.86-1.04)	67.06	55	0.82 (0.62-1.07)
64	Financial service activities, except insurance and pension funding	2,054.73	2,070	1.01 (0.96-1.05)	1,910.52	1,761	0.92 (0.88-0.97)	298.52	309	1.04 (0.92-1.16)

65	Insurance and pension funding	257.91	250	0.97 (0.86-1.10)	241.47	196	0.81 (0.70-0.93)	38.13	54	1.42 (1.06-1.85)
66	Activities auxiliary to financial service and insurance activities	118.04	96	0.81 (0.68-0.98)	102.07	81	0.79 (0.63-0.99)	20.16	15	0.74 (0.42-1.23)
68	Real estate activities	5,248.73	5,337	1.02 (0.99-1.04)	4,439.61	4,590	1.03 (1.00-1.06)	745.64	747	1.00 (0.93-1.08)
70	Research and development	46.25	44	0.95 (0.71-1.30)	42.86	40	0.93 (0.67-1.27)	6.36	4	0.63 (0.17-1.61)
71	Professional services	1,111.35	1,020	0.92 (0.87-0.97)	923.34	846	0.92 (0.86-0.98)	207.71	174	0.84 (0.72-0.97)
72	Architectural, engineering and other scientific technical services	988.12	1,068	1.08 (1.02-1.15)	1,020.56	1,001	0.98 (0.92-1.04)	71.91	67	0.93 (0.72-1.18)
73	Other professional, scientific and technical services	117.04	123	1.05 (0.88-1.27)	98.62	100	1.01 (0.83-1.23)	19.60	23	1.17 (0.74-1.76)
74	Business facilities management and landscape services	1,598.18	1,479	0.93 (0.88-0.97)	1,014.79	1,114	1.10 (1.04-1.17)	353.07	363	1.03 (0.93-1.14)
75	Business support services	3,815.57	3,713	0.97 (0.94-1.00)	2,742.75	2,883	1.05 (1.01-1.09)	783.29	830	1.06 (0.99-1.13)
76	Rental and leasing activities; except real estate	172.32	189	1.10 (0.94-1.28)	165.96	171	1.03 (0.88-1.20)	18.10	18	0.99 (0.59-1.57)
84	Public administration and defence; compulsory social security	700.04	749	1.07 (0.99-1.15)	590.55	646	1.09 (1.01-1.18)	111.35	103	0.93 (0.76-1.12)
85	Education	2,328.99	1,652	0.71 (0.68-0.74)	752.48	686	0.91 (0.84-0.98)	995.91	966	0.97 (0.91-1.03)
86	Human health activities	2,562.50	2,070	0.81 (0.78-0.84)	1,332.87	1,231	0.92 (0.87-0.98)	839.21	839	1.00 (0.93-1.07)
87	Social work activities	1,798.76	1,239	0.69 (0.66-0.72)	541.02	534	0.99 (0.91-1.07)	719.16	705	0.98 (0.91-1.06)
90	Creative, arts and recreation related services	73.63	67	0.91 (0.72-1.16)	53.88	53	0.98 (0.74-1.29)	16.02	14	0.87 (0.48-1.47)
91	Sports activities and amusement activities	1,085.88	1,132	1.04 (0.98-1.11)	827.84	898	1.08 (1.01-1.16)	198.25	234	1.18 (1.03-1.34)
94	Membership organizations	2,037.21	1,861	0.91 (0.87-0.95)	1,580.42	1,495	0.95 (0.90-1.00)	369.76	366	0.99 (0.89-1.10)
95	Maintenance and repair services of personal and household goods	447.86	498	1.11 (1.01-1.22)	450.75	463	1.03 (0.94-1.13)	38.16	35	0.92 (0.64-1.28)
96	Other personal services activities	1,739.66	1,697	0.98 (0.93-1.02)	1,242.99	1,349	1.09 (1.03-1.14)	364.74	348	0.95 (0.86-1.06)
98	Undifferentiated goods-and services-producing activities of private households for own use	588.06	628	1.07 (0.98-1.16)	507.31	539	1.06 (0.97-1.16)	80.72	89	1.10 (0.89-1.36)
99	Activities of extraterritorial organizations and bodies	191.22	209	1.09 (0.95-1.27)	175.06	187	1.07 (0.92-1.23)	22.44	22	0.98 (0.61-1.48)
	Non-confirmed	13,598.26	13,531	1.00 (0.98-1.01)	11,735.86	11,379	0.97 (0.95-0.99)	2,122.95	2,152	1.01 (0.97-1.06)

Supplementary Table S2. Age-standardized incidence ratio (SIR) and 95% confidence interval (CI) of malignant neoplasms of lip, oral cavity and pharynx according to divisions of the Korean Standard Industrial Classification (KSIC).

KSIC		Total workers			Male workers			Female workers		
Divisions	Description	Expected cases	Observed cases	SIR (95% CI)	Expected cases	Observed cases	SIR (95% CI)	Expected cases	Observed cases	SIR (95% CI)
01	Agriculture	6.18	4	0.65 (0.30-1.73)	5.36	4	0.75 (0.20-1.91)	0.78	0	0.00 (0.00-0.00)
02	Forestry	1.39	3	2.15 (0.48-5.12)	1.45	1	1.38 (0.17-4.99)	0.10	2	9.72 (0.25-54.15)
03	Fishing and aquaculture	4.10	4	0.98 (0.39-3.51)	3.58	4	1.12 (0.30-2.86)	0.59	0	0.00 (0.00-0.00)
05	Mining of coal, crude petroleum and natural gas	5.70	5	0.88 (0.29-2.05)	6.07	4	0.66 (0.18-1.69)	0.34	1	2.94 (0.07-16.38)
06	Mining of metal ores	0.51	1	1.96 (0.05-10.92)	0.54	1	1.86 (0.05-10.34)	0.03	0	0.00 (0.00-0.00)
07	Mining of non-metallic minerals, except fuel	1.93	2	1.04 (0.13-3.74)	2.10	2	0.95 (0.12-3.44)	0.10	0	0.00 (0.00-0.00)
08	Mining support service activities	0.54	1	1.85 (0.05-10.32)	0.54	1	1.85 (0.05-10.32)	0.05	0	0.00 (0.00-0.00)
10	Manufacture of food products	44.72	42	0.94 (0.70-1.29)	27.12	38	1.40 (0.99-1.92)	10.05	4	0.40 (0.11-1.02)
11	Manufacture of beverages	1.97	3	1.52 (0.42-12.91)	1.82	2	1.10 (0.13-3.58)	0.22	1	4.63 (0.12-25.77)
12	Manufacture of tobacco products	0.15	0	0.00 (0.00-0.00)	0.14	0	0.00 (0.00-0.00)	0.02	0	0.00 (0.00-0.00)
13	Manufacture of textiles, except apparel	40.40	34	0.84 (0.62-1.18)	30.15	29	0.96 (0.64-1.38)	7.42	5	0.67 (0.22-1.57)
14	Manufacture of wearing apparel, clothing accessories and fur articles	11.14	11	0.99 (0.55-1.97)	6.13	9	1.47 (0.67-2.79)	2.97	2	0.67 (0.08-2.43)
15	Manufacture of leather, luggage and footwear	7.34	3	0.41 (0.20-0.99)	5.37	3	0.56 (0.12-1.63)	1.35	0	0.00 (0.00-0.00)
16	Manufacture of wood and of products of wood and cork; except furniture	11.03	11	1.00 (0.56-2.00)	10.59	10	0.94 (0.45-1.74)	1.06	1	0.94 (0.02-5.24)
17	Manufacture of pulp, paper and paper products	12.20	10	0.82 (0.47-1.58)	11.80	8	0.68 (0.29-1.34)	1.14	2	1.75 (0.21-6.32)
18	Printing and reproduction of recorded media	27.60	31	1.12 (0.78-1.70)	23.29	23	0.99 (0.63-1.48)	4.71	8	1.70 (0.73-3.35)
19	Manufacture of coke, briquettes and refined petroleum products	3.25	3	0.92 (0.33-4.12)	3.66	3	0.82 (0.17-2.39)	0.12	0	0.00 (0.00-0.00)
20	Manufacture of chemicals and chemical products; except pharmaceuticals and medicinal chemicals	44.15	48	1.09 (0.80-1.09)	42.22	43	1.02 (0.74-1.37)	4.55	5	1.10 (0.36-2.56)
21	Manufacture of pharmaceuticals, medicinal chemical and botanical products	3.26	3	0.92 (0.33-4.08)	2.73	2	0.73 (0.09-2.65)	0.53	1	1.90 (0.05-10.98)
22	Manufacture of rubber and plastics products	16.28	17	1.04 (0.65-1.82)	14.40	13	0.90 (0.48-1.54)	2.07	4	1.93 (0.53-4.95)
23	Manufacture of other non-metallic mineral products	18.30	29	1.58 (1.01-2.66)	18.37	25	1.36 (0.88-2.01)	1.46	4	2.74 (0.75-7.01)
24	Manufacture of basic metals	42.79	48	1.12 (0.83-1.55)	45.51	47	1.03 (0.76-1.37)	2.47	1	0.40 (0.01-2.24)
25	Manufacture of fabricated metal products, except machinery and furniture	40.24	36	0.89 (0.66-1.25)	39.62	31	0.78 (0.53-1.11)	3.58	5	1.40 (0.45-3.26)
26	Manufacture of electronic components, computer; visual, sounding and communication equipment	61.23	57	0.93 (0.73-1.22)	48.44	44	0.91 (0.66-1.22)	11.38	13	1.14 (0.61-1.95)

27	Manufacture of medical, precision and optical instruments, watches and clocks	6.11	2	0.33 (0.15-0.88)	5.05	2	0.40 (0.05-1.43)	1.00	0	0.00 (0.00-0.00)
28	Manufacture of electrical equipment	25.13	32	1.27 (0.86-1.97)	23.25	29	1.25 (0.84-1.79)	2.94	3	1.02 (0.21-2.98)
29	Manufacture of other machinery and equipment	55.40	61	1.10 (0.85-1.46)	55.38	55	0.99 (0.75-1.29)	4.72	6	1.27 (0.47-2.77)
30	Manufacture of motor vehicles, trailers and semitrailers	84.44	85	1.01 (0.81-1.26)	82.21	76	0.92 (0.73-1.16)	7.79	9	1.19 (0.54-2.25)
31	Manufacture of other transport equipment	43.42	41	0.94 (0.70-1.30)	47.42	39	0.82 (0.58-1.12)	2.03	2	0.98 (0.12-3.55)
32	Manufacture of furniture	8.22	7	0.85 (0.44-1.95)	7.55	6	0.79 (0.29-1.73)	0.92	1	1.09 (0.03-6.06)
33	Other manufacturing	285.84	271	0.95 (0.84-1.07)	259.75	239	0.92 (0.81-1.04)	34.73	32	0.92 (0.63-1.30)
35	Electricity, gas, steam and air conditioning supply	28.01	34	1.21 (0.84-1.83)	31.10	33	1.06 (0.73-1.49)	1.28	1	0.78 (0.02-4.35)
36	Water supply	1.87	1	0.54 (0.14-4.95)	2.05	1	0.49 (0.01-2.72)	0.10	0	0.00 (0.00-0.00)
37	Sewage, wastewater, human and animal waste treatment services	1.40	2	1.43 (0.32-22.93)	1.27	2	1.58 (0.19-5.71)	0.15	0	0.00 (0.00-0.00)
38	Waste collection, treatment and disposal activities; materials recovery	8.02	4	0.50 (0.25-1.15)	7.89	4	0.51 (0.14-1.30)	0.07	0	0.00 (0.00-0.00)
41	General construction	150.28	157	1.04 (0.89-1.23)	159.35	149	0.94 (0.79-1.10)	9.45	8	0.85 (0.37-1.67)
42	Specialized construction activities	92.60	119	1.29 (1.05-1.59)	97.12	113	1.16 (0.96-1.40)	6.43	6	0.93 (0.34-2.03)
45	Sale of motor vehicles and parts	12.04	14	1.16 (0.67-2.25)	11.98	13	1.08 (0.58-1.86)	1.07	1	0.93 (0.02-5.18)
46	Wholesale trade on own account or on a fee or contract basis	150.44	172	1.14 (0.97-1.35)	126.22	149	1.18 (1.00-1.39)	23.95	23	0.96 (0.61-1.44)
47	Retail trade, except motor vehicles and motorcycles	151.58	148	0.98 (0.83-1.15)	118.65	116	0.98 (0.81-1.17)	28.16	32	1.14 (0.78-1.60)
49	Land transport and transport via pipelines	42.39	48	1.13 (0.84-1.57)	47.27	46	0.97 (0.71-1.30)	1.87	2	1.07 (0.13-3.87)
50	Water transport	12.09	14	1.16 (0.66-2.24)	13.85	13	0.94 (0.50-1.61)	0.46	1	2.18 (0.06-12.17)
51	Air transport	1.68	4	2.38 (0.60-26.49)	1.72	4	2.32 (0.63-5.94)	0.16	0	0.00 (0.00-0.00)
52	Warehousing and support activities for transportation	160.76	210	1.31 (1.12-1.53)	182.96	204	1.12 (0.97-1.28)	5.99	6	1.00 (0.37-2.18)
55	Accommodation	16.50	17	1.03 (0.64-1.79)	10.01	10	1.00 (0.48-1.84)	3.65	7	1.92 (0.77-3.95)
56	Food and beverage service activities	61.50	37	0.60 (0.47-0.79)	21.68	22	1.01 (0.64-1.54)	19.08	15	0.79 (0.44-1.30)
59	Motion picture, video and television program production, sound recording and music publishing activities	2.34	2	0.86 (0.26-5.63)	2.17	2	0.92 (0.11-3.33)	0.33	0	0.00 (0.00-0.00)
60	Broadcasting activities	1.80	0	0.00 (0.00-0.00)	1.10	0	0.00 (0.00-0.00)	0.55	0	0.00 (0.00-0.00)
61	Postal activities and telecommunications	21.69	25	1.15 (0.76-1.85)	22.61	25	1.11 (0.72-1.63)	1.69	0	0.00 (0.00-0.00)
62	Computer programming, consultancy and related activities	15.16	18	1.19 (0.72-2.11)	13.91	16	1.15 (0.66-1.87)	2.24	2	0.89 (0.11-3.23)
63	Information service activities	22.34	23	1.03 (0.68-1.64)	20.22	20	0.99 (0.60-1.53)	3.39	3	0.88 (0.18-2.59)
64	Financial service activities, except insurance and pension funding	84.12	91	1.08 (0.87-1.36)	75.14	81	1.08 (0.86-1.34)	14.66	10	0.68 (0.33-1.25)

65	Insurance and pension funding	10.71	11	1.03 (0.57-2.08)	9.62	8	0.83 (0.36-1.64)	1.90	3	1.58 (0.33-4.61)
66	Activities auxiliary to financial service and insurance activities	4.89	5	1.02 (0.43-3.20)	4.11	5	1.22 (0.39-2.84)	0.93	0	0.00 (0.00-0.00)
68	Real estate activities	173.88	162	0.93 (0.80-1.09)	150.84	140	0.93 (0.78-1.10)	20.68	22	1.06 (0.67-1.61)
70	Research and development	1.91	3	1.57 (0.42-14.04)	1.72	3	1.75 (0.36-5.11)	0.30	0	0.00 (0.00-0.00)
71	Professional services	43.55	47	1.08 (0.80-1.49)	34.60	42	1.21 (0.87-1.64)	9.26	5	0.54 (0.18-1.26)
72	Architectural, engineering and other scientific technical services	36.64	37	1.01 (0.73-1.44)	37.98	36	0.95 (0.66-1.31)	2.93	1	0.34 (0.01-1.90)
73	Other professional, scientific and technical services	4.77	4	0.84 (0.35-2.67)	4.00	4	1.00 (0.27-2.56)	0.82	0	0.00 (0.00-0.00)
74	Business facilities management and landscape services	50.90	49	0.96 (0.73-1.29)	33.54	40	1.19 (0.85-1.62)	8.62	9	1.06 (0.48-2.01)
75	Business support services	133.97	117	0.87 (0.74-1.04)	97.08	86	0.89 (0.71-1.09)	25.17	31	1.23 (0.84-1.75)
76	Rental and leasing activities; except real estate	6.66	12	1.80 (0.86-4.61)	6.44	8	1.24 (0.54-2.45)	0.72	4	5.53 (1.51-14.15)
84	Public administration and defence; compulsory social security	25.08	30	1.20 (0.81-1.85)	21.14	27	1.28 (0.84-1.86)	3.93	3	0.76 (0.16-2.23)
85	Education	92.19	69	0.75 (0.61-0.93)	27.91	30	1.07 (0.73-1.53)	38.81	39	1.00 (0.71-1.37)
86	Human health activities	100.31	102	1.02 (0.84-1.25)	49.85	61	1.22 (0.94-1.57)	33.21	41	1.23 (0.89-1.67)
87	Social work activities	66.74	51	0.76 (0.60-0.99)	20.23	31	1.53 (1.04-2.18)	24.40	20	0.82 (0.50-1.27)
90	Creative, arts and recreation related services	2.93	2	0.68 (0.23-3.39)	2.13	2	0.94 (0.11-3.39)	0.64	0	0.00 (0.00-0.00)
91	Sports activities and amusement activities	37.78	34	0.90 (0.66-1.27)	29.31	28	0.96 (0.63-1.38)	6.13	6	0.98 (0.36-2.13)
94	Membership organizations	72.14	60	0.83 (0.66-1.06)	56.70	51	0.90 (0.67-1.18)	11.91	9	0.76 (0.35-1.43)
95	Maintenance and repair services of personal and household goods	17.96	12	0.67 (0.42-1.13)	18.11	8	0.44 (0.19-0.87)	1.62	4	2.46 (0.67-6.31)
96	Other personal services activities	62.29	64	1.03 (0.80-1.34)	44.93	56	1.25 (0.94-1.62)	12.01	8	0.67 (0.29-1.31)
98	Undifferentiated goods-and services-producing activities of private households for own use	18.77	14	0.75 (0.48-1.24)	16.36	14	0.86 (0.47-1.44)	2.33	0	0.00 (0.00-0.00)
99	Activities of extraterritorial organizations and bodies	6.51	3	0.46 (0.22-1.20)	6.08	2	0.33 (0.04-1.19)	0.68	1	1.48 (0.04-8.24)
	Non-confirmed	505.88	497	0.98 (0.90-1.07)	434.49	408	0.94 (0.85-1.03)	79.77	89	1.12 (0.90-1.37)

Supplementary Table S3. Age-standardized incidence ratio (SIR) and 95% confidence interval (CI) of malignant neoplasm of oesophagus according to divisions of the Korean Standard Industrial Classification (KSIC).

KSIC		Total workers			Male workers			Female workers		
Divisions	Description	Expected cases	Observed cases	SIR (95% CI)	Expected cases	Observed cases	SIR (95% CI)	Expected cases	Observed cases	SIR (95% CI)
01	Agriculture	3.75	6	1.60 (0.59-3.48)	3.54	4	1.13 (0.31-2.90)	0.13	2	15.83 (1.92-57.20)
02	Forestry	0.95	3	3.16 (0.65-9.23)	1.12	3	2.69 (0.55-7.85)	0.01	0	0.00 (0.00-0.00)
03	Fishing and aquaculture	2.38	0	0.00 (0.00-0.00)	2.48	0	0.00 (0.00-0.00)	0.07	0	0.00 (0.00-0.00)
05	Mining of coal, crude petroleum and natural gas	3.61	5	1.39 (0.52-5.58)	4.25	5	1.18 (0.38-2.75)	0.05	0	0.00 (0.00-0.00)
06	Mining of metal ores	0.31	1	3.21 (0.08-17.86)	0.36	1	2.75 (0.07-15.35)	0.00	0	0.00 (0.00-0.00)
07	Mining of non-metallic minerals, except fuel	1.31	2	1.53 (0.19-5.53)	1.57	2	1.28 (0.15-4.61)	0.02	0	0.00 (0.00-0.00)
08	Mining support service activities	0.36	0	0.00 (0.00-10.16)	0.40	0	0.00 (0.00-0.00)	0.01	0	0.00 (0.00-0.00)
10	Manufacture of food products	24.30	11	0.45 (0.23-0.81)	15.03	11	0.73 (0.37-1.31)	1.51	0	0.00 (0.00-0.00)
11	Manufacture of beverages	0.97	0	0.00 (0.00-0.00)	0.96	0	0.00 (0.00-0.00)	0.03	0	0.00 (0.00-0.00)
12	Manufacture of tobacco products	0.07	0	0.00 (0.00-0.00)	0.07	0	0.00 (0.00-0.00)	0.00	0	0.00 (0.00-0.00)
13	Manufacture of textiles, except apparel	21.94	14	0.64 (0.35-1.072)	18.12	14	0.77 (0.42-1.30)	1.00	0	0.00 (0.00-0.00)
14	Manufacture of wearing apparel, clothing accessories and fur articles	5.76	2	0.35 (0.04-1.25)	3.42	2	0.59 (0.07-2.12)	0.39	0	0.00 (0.00-0.00)
15	Manufacture of leather, luggage and footwear	4.20	4	0.95 (0.26-2.44)	3.36	4	1.19 (0.32-3.04)	0.20	0	0.00 (0.00-0.00)
16	Manufacture of wood and of products of wood and cork; except furniture	6.57	8	1.22 (0.58-3.14)	7.00	8	1.14 (0.49-2.25	0.15	0	0.00 (0.00-0.00)
17	Manufacture of pulp, paper and paper products	6.62	1	0.15 (0.07-0.39)	7.03	1	0.14 (0.00-0.79)	0.16	0	0.00 (0.00-0.00)
18	Printing and reproduction of recorded media	12.47	16	1.28 (0.74-2.45)	12.47	16	1.28 (0.73-2.08)	0.43	0	0.00 (0.00-0.00)
19	Manufacture of coke, briquettes and refined petroleum products	1.35	1	0.74 (0.16-12.85)	1.73	1	0.58 (0.01-3.23)	0.01	0	0.00 (0.00-0.00)
20	Manufacture of chemicals and chemical products; except pharmaceuticals and medicinal chemicals	20.95	26	1.24 (0.81-2.01)	22.26	25	1.12 (0.73-1.66)	0.53	1	1.88 (0.05-10.47)
21	Manufacture of pharmaceuticals, medicinal chemical and botanical products	1.38	0	0.00 (0.00-0.00)	1.29	0	0.00 (0.00-0.00)	0.05	0	0.00 (0.00-0.00)
22	Manufacture of rubber and plastics products	8.44	8	0.95 (0.49-2.14)	8.25	8	0.97 (0.42-1.91)	0.27	0	0.00 (0.00-0.00)
23	Manufacture of other non-metallic mineral products	10.16	9	0.89 (0.48-1.84)	11.25	9	0.80 (0.37-1.52)	0.20	0	0.00 (0.00-0.00)
24	Manufacture of basic metals	22.83	22	0.96 (0.64-1.52)	26.97	22	0.82 (0.51-1.23)	0.32	0	0.00 (0.00-0.00)
25	Manufacture of fabricated metal products, except machinery and furniture	20.33	24	1.18 (0.77-1.92)	22.27	24	1.08 (0.69-1.60)	0.45	0	0.00 (0.00-0.00)
26	Manufacture of electronic components, computer; visual, sounding and communication equipment	20.96	16	0.76 (0.50-1.23)	18.62	16	0.86 (0.49-1.40)	1.00	0	0.00 (0.00-0.00)

27	Manufacture of medical, precision and optical instruments, watches and clocks	2.59	3	1.16 (0.37-6.68)	2.42	3	1.24 (0.26-3.62)	0.10	0	0.00 (0.00-0.00)
28	Manufacture of electrical equipment	11.73	10	0.85 (0.49-1.66)	12.23	9	0.74 (0.34-1.40)	0.33	1	3.03 (0.08-16.88)
29	Manufacture of other machinery and equipment	26.76	22	0.82 (0.56-1.25)	29.99	22	0.73 (0.46-1.11)	0.54	0	0.00 (0.00-0.00)
30	Manufacture of motor vehicles, trailers and semitrailers	36.14	23	0.64 (0.46-0.91)	38.15	23	0.60 (0.38-0.90)	0.94	0	0.00 (0.00-0.00)
31	Manufacture of other transport equipment	22.96	24	1.05 (0.70-1.65)	28.05	23	0.82 (0.52-1.23)	0.24	1	4.15 (0.11-23.13)
32	Manufacture of furniture	4.28	2	0.47 (0.19-1.62)	4.30	2	0.46 (0.06-1.68)	0.13	0	0.00 (0.00-0.00)
33	Other manufacturing	138.83	121	0.87 (0.74-1.04)	139.73	117	0.84 (0.69-1.00)	4.18	4	0.96 (0.26-2.45)
35	Electricity, gas, steam and air conditioning supply	14.63	17	1.16 (0.70-2.09)	18.46	17	0.92 (0.54-1.47)	0.11	0	0.00 (0.00-0.00)
36	Water supply	0.95	1	1.05 (0.03-6.86)	0.85	1	0.85 (0.02-4.71)	0.01	0	0.00 (0.00-0.00)
37	Sewage, wastewater, human and animal waste treatment services	1.01	1	0.99 (0.18-38.37)	0.96	1	1.04 (0.03-5.79)	0.03	0	0.00 (0.00-0.00)
38	Waste collection, treatment and disposal activities; materials recovery	5.26	7	1.33 (0.58-3.96)	5.65	7	1.24 (0.50-2.55)	0.12	0	0.00 (0.00-0.00)
41	General construction	83.45	119	1.43 (1.15-1.79)	98.62	116	1.18 (0.97-1.41)	1.17	3	2.57 (0.53-7.51)
42	Specialized construction activities	49.87	83	1.66 (1.26-2.24)	58.99	79	1.34 (1.06-1.67)	0.72	4	5.59 (1.52-14.31)
45	Sale of motor vehicles and parts	5.48	9	1.64 (0.73-4.75)	6.15	8	1.30 (0.56-2.56)	0.11	1	8.75 (0.22-48.77)
46	Wholesale trade on own account or on a fee or contract basis	74.94	87	1.16 (0.93-1.48)	72.03	84	1.17 (0.93-1.44)	2.67	3	1.12 (0.23-3.28)
47	Retail trade, except motor vehicles and motorcycles	72.90	74	1.02 (0.81-1.30)	66.19	70	1.06 (0.82-1.34)	3.04	4	1.32 (0.36-3.37)
49	Land transport and transport via pipelines	26.03	27	1.04 (0.71-1.59)	32.42	27	0.83 (0.55-1.21)	0.21	0	0.00 (0.00-0.00)
50	Water transport	8.52	7	0.82 (0.43-1.85)	10.88	7	0.64 (0.26-1.33)	0.04	0	0.00 (0.00-0.00)
51	Air transport	0.77	1	1.30 (0.19-134.69)	0.94	1	1.07 (0.03-5.95)	0.01	0	0.00 (0.00-0.00)
52	Warehousing and support activities for transportation	105.35	129	1.22 (1.02-1.46)	133.31	128	0.96 (0.80-1.14)	0.64	1	1.56 (0.04-8.68)
55	Accommodation	9.39	4	0.43 (0.23-0.91)	5.67	4	0.71 (0.19-1.81)	0.58	0	0.00 (0.00-0.00)
56	Food and beverage service activities	36.30	24	0.66 (0.48-0.94)	12.28	16	1.30 (0.74-2.12)	3.12	8	2.56 (1.11-5.05)
59	Motion picture, video and television program production, sound recording and music publishing activities	0.86	2	2.31 (0.28-8.36)	0.97	2	2.06 (0.25-7.44)	0.02	0	0.00 (0.00-0.00)
60	Broadcasting activities	0.65	1	1.53 (0.20-332.85)	0.52	1	1.94 (0.05-10.80)	0.04	0	0.00 (0.00-0.00)
61	Postal activities and telecommunications	9.09	4	0.44 (0.23-0.96)	11.00	4	0.36 (0.10-0.93)	0.13	0	0.00 (0.00-0.00)
62	Computer programming, consultancy and related activities	3.87	2	0.52 (0.06-1.86)	4.38	2	0.46 (0.06-1.65)	0.11	0	0.00 (0.00-0.00)
63	Information service activities	6.13	7	1.14 (0.53-3.07)	6.72	6	0.89 (0.33-1.94)	0.19	1	5.17 (0.13-28.79)
64	Financial service activities, except insurance and pension funding	32.75	26	0.79 (0.52-1.16)	37.71	26	0.69 (0.45-1.01)	0.83	0	0.00 (0.00-0.00)

65	Insurance and pension funding	3.74	2	0.53 (0.20-2.08)	4.40	2	0.45 (0.06-1.64)	3.74	0	0.00 (0.00-0.00)
66	Activities auxiliary to financial service and insurance activities	1.88	2	1.06 (0.13-3.84)	1.94	2	1.03 (0.13-3.73)	0.07	0	0.00 (0.00-0.00)
68	Real estate activities	133.36	157	1.18 (0.99-1.41)	124.46	154	1.23 (1.04-1.44)	4.46	3	0.67 (0.14-1.97)
70	Research and development	0.78	0	0.00 (0.00-0.00)	0.86	0	0.00 (0.00-0.00)	0.02	0	0.00 (0.00-0.00)
71	Professional services	20.84	20	0.96 (0.63-1.55)	21.49	20	0.93 (0.57-1.44)	0.70	0	0.00 (0.00-0.00)
72	Architectural, engineering and other scientific technical services	20.64	23	1.11 (0.71-1.67)	24.51	23	0.94 (0.59-1.41)	0.28	0	0.00 (0.00-0.00)
73	Other professional, scientific and technical services	2.03	3	1.47 (0.30-4.31)	1.99	3	1.51 (0.31-4.41)	0.07	0	0.00 (0.00-0.00)
74	Business facilities management and landscape services	43.05	44	1.02 (0.74-1.37)	30.20	42	1.39 (1.00-1.88)	2.31	2	0.87 (0.11-3.13)
75	Business support services	89.46	72	0.80 (0.65-1.00)	74.24	69	0.93 (0.72-1.18)	3.99	3	0.75 (0.16-2.20)
76	Rental and leasing activities; except real estate	3.35	2	0.60 (0.22-2.58)	3.72	2	0.54 (0.07-1.94)	0.07	0	0.00 (0.00-0.00)
84	Public administration and defence; compulsory social security	15.21	28		15.05	28	1.86 (1.24-2.69)	0.48	0	0.00 (0.00-0.00)
85	Education	40.58	14	0.35 (0.25-0.48)	18.31	12	0.66 (0.34-1.14)	3.69	2	0.54 (0.07-1.96)
86	Human health activities	48.43	35	0.72 (0.55-0.98)	30.57	32	1.05 (0.72-1.48)	3.37	3	0.89 (0.18-2.60)
87	Social work activities	36.98	11	0.30 (0.22-0.42)	12.94	7	0.54 (0.22-1.11)	3.26	4	1.23 (0.33-3.15)
90	Creative, arts and recreation related services	1.39	3	2.15 (0.48-35.04)	1.17	3	2.56 (0.53-7.47)	0.07	0	0.00 (0.00-0.00)
91	Sports activities and amusement activities	25.78	39	1.51 (1.03-2.32)	22.17	39	1.76 (1.25-2.40)	1.07	0	0.00 (0.00-0.00)
94	Membership organizations	45.93	35	0.76 (0.57-1.04)	40.40	34	0.84 (0.58-1.18)	1.86	1	0.54 (0.01-2.99)
95	Maintenance and repair services of personal and household goods	7.74	7	0.90 (0.36-1.86)	9.04	7	0.77 (0.31-1.60)	0.14	0	0.00 (0.00-0.00)
96	Other personal services activities	38.63	46	1.19 (0.87-1.59)	31.94	43	1.35 (0.97-1.81)	1.75	3	1.71 (0.35-5.01)
98	Undifferentiated goods-and services-producing activities of private households for own use	15.88	12	0.76 (0.39-1.32)	15.65	12	0.77 (0.40-1.34)	0.46	0	0.00 (0.00-0.00)
99	Activities of extraterritorial organizations and bodies	4.53	9	1.99 (0.91-3.77)	4.67	9	1.93 (0.88-3.66)	0.12	0	0.00 (0.00-0.00)
	Non-confirmed	270.37	284	1.05 (0.93-1.19)	275.60	275	1.00 (0.88-1.12)	8.31	9	1.08 (0.50-2.06)

Supplementary Table S4. Age-standardized incidence ratio (SIR) and 95% confidence interval (CI) of malignant neoplasm of stomach according to divisions of the Korean Standard Industrial Classification (KSIC).

KSIC		Total workers			Male workers			Female workers		
Divisions	Description	Expected cases	Observed cases	SIR (95% CI)	Expected cases	Observed cases	SIR (95% CI)	Expected cases	Observed cases	SIR (95% CI)
01	Agriculture	62.09	52	0.84 (0.65-1.09)	52.12	48	0.92 (0.68-1.22)	62.09	52	0.44 (0.12-1.12)
02	Forestry	14.47	13	0.90 (0.54-1.62)	14.82	13	0.88 (0.47-1.50)	1.14	0	0.00 (0.00-0.00)
03	Fishing and aquaculture	41.09	47	1.14 (0.84-1.59)	35.54	42	1.18 (0.85-1.60)	6.48	5	0.77 (0.25-1.80)
05	Mining of coal, crude petroleum and natural gas	57.83	79	1.37 (1.06-1.80)	60.30	72	1.19 (0.93-1.50)	3.86	7	1.82 (0.73-3.74)
06	Mining of metal ores	5.12	4	0.78 (0.34-2.37)	5.25	4	0.76 (0.21-1.95)	0.36	0	0.00 (0.00-0.00)
07	Mining of non-metallic minerals, except fuel	20.01	27	1.35 (0.87-2.21)	21.26	26	1.22 (0.80-1.79)	1.14	1	0.87 (0.02-4.87)
08	Mining support service activities	5.59	4	0.72 (0.32-2.04)	5.49	3	0.55 (0.11-1.60)	0.51	1	1.95 (0.05-10.84)
10	Manufacture of food products	438.33	372	0.85 (0.77-0.93)	251.83	248	0.98 (0.87-1.12)	115.43	124	1.07 (0.89-1.28)
11	Manufacture of beverages	18.89	22	1.16 (0.74-1.94)	16.78	21	1.25 (0.77-1.91)	2.39	1	0.42 (0.01-2.33)
12	Manufacture of tobacco products	1.41	0	0.00 (0.00-0.00)	1.22	0	0.00 (0.00-0.00)	0.18	0	0.00 (0.00-0.00)
13	Manufacture of textiles, except apparel	397.44	366	0.92 (0.83-1.02)	288.33	279	0.97 (0.86-1.09)	83.35	87	1.04 (0.84-1.29)
14	Manufacture of wearing apparel, clothing accessories and fur articles	107.89	70	0.65 (0.54-0.79)	57.39	46	0.80 (0.59-1.07)	32.78	24	0.73 (0.47-1.09)
15	Manufacture of leather, luggage and footwear	72.86	67	0.92 (0.73-1.17)	51.77	52	1.00 (0.75-1.32)	15.36	15	0.98 (0.55-1.61)
16	Manufacture of wood and of products of wood and cork; except furniture	110.47	120	1.09 (0.90-1.32)	103.20	109	1.06 (0.87-1.27)	12.17	11	0.90 (0.45-1.62)
17	Manufacture of pulp, paper and paper products	120.00	160	1.33 (1.12-1.61)	112.53	139	1.24 (1.04-1.46)	13.00	21	1.62 (1.00-2.48)
18	Printing and reproduction of recorded media	259.57	243	0.94 (0.83-1.06)	216.27	190	0.88 (0.76-1.01)	47.807	53	1.11 (0.83-1.45)
19	Manufacture of coke, briquettes and refined petroleum products	30.91	37	1.20 (0.84-1.76)	33.85	36	1.06 (0.74-1.47)	1.12	1	0.89 (0.02-4.98)
20	Manufacture of chemicals and chemical products; except pharmaceuticals and medicinal chemicals	418.32	457	1.09 (0.99-1.21)	387.22	406	1.05 (0.95-1.16)	48.61	51	1.05 (0.78-1.38)
21	Manufacture of pharmaceuticals, medicinal chemical and botanical products	30.04	25	0.83 (0.58-1.23)	24.22	18	0.74 (0.44-1.17)	5.50	7	1.27 (0.51-2.62)
22	Manufacture of rubber and plastics products	157.97	157	0.99 (0.85-1.17)	134.71	127	0.94 (0.79-1.12)	23.41	30	1.28 (0.86-1.83)
23	Manufacture of other non-metallic mineral products	180.05	200	1.11 (0.96-1.29)	175.23	181	1.03 (0.89-1.19)	16.47	19	1.15 (0.69-1.80)
24	Manufacture of basic metals	418.12	495	1.18 (1.08-1.31)	431.64	464	1.07 (0.98-1.18)	27.56	31	1.12 (0.76-1.60)
25	Manufacture of fabricated metal products, except machinery and furniture	387.25	432	1.12 (1.01-1.24)	368.74	391	1.06 (0.96-1.17)	39.51	41	1.04 (0.74-1.41)
26	Manufacture of electronic components, computer; visual, sounding and communication equipment	541.52	484	0.89 (0.82-0.97)	405.63	361	0.89 (0.80-0.99)	117.67	123	1.05 (0.87-1.25)

27	Manufacture of medical, precision and optical instruments, watches and clocks	56.62	56	0.99 (0.76-1.31)	44.91	48	1.07 (0.79-1.42)	10.67	8	0.75 (0.32-1.48)
28	Manufacture of electrical equipment	237.90	226	0.95 (0.84-1.08)	212.60	200	0.94 (0.82-1.08)	32.06	26	0.81 (0.53-1.19)
29	Manufacture of other machinery and equipment	528.10	565	1.07 (0.98-1.17)	510.8	517	1.01 (0.93-1.10)	51.21	48	0.94 (0.69-1.24)
30	Manufacture of motor vehicles, trailers and semitrailers	795.17	803	1.01 (0.94-1.08)	741.00	717	0.97 (0.90-1.04)	86.44	86	1.02 (0.81-1.26)
31	Manufacture of other transport equipment	418.52	520	1.24 (1.13-1.37)	443.94	502	1.13 (1.03-1.23)	22.17	18	0.81 (0.48-1.28)
32	Manufacture of furniture	79.81	93	1.17 (0.94-1.47)	70.77	77	1.09 (0.86-1.36)	10.37	16	1.54 (0.88-2.51)
33	Other manufacturing	2,719.96	2,846	1.05 (1.01-1.09)	2,389.06	2,480	1.04 (1.00-1.08)	374.97	366	0.98 (0.88-1.08)
35	Electricity, gas, steam and air conditioning supply	272.52	323	1.19 (1.05-1.34)	296.02	311	1.05 (0.94-1.17)	13.14	12	0.91 (0.47-1.59)
36	Water supply	18.10	18	0.99 (0.63-1.68)	19.41	18	0.93 (0.55-1.47)	1.01	0	0.00 (0.00-0.00)
37	Sewage, wastewater, human and animal waste treatment services	14.76	15	1.02 (0.61-1.83)	12.87	14	1.09 (0.59-1.83)	1.87	1	0.53 (0.01-2.97)
38	Waste collection, treatment and disposal activities; materials recovery	82.37	98	1.19 (0.96-1.50)	78.88	90	1.14 (0.92-1.40)	8.02	8	1.00 (0.43-1.97)
41	General construction	1,485.50	1,568	1.06 (1.00-1.11)	1,536.90	1,458	0.95 (0.90-1.00	101.50	110	1.08 (0.89-1.31)
42	Specialized construction activities	910.48	1,025	1.13 (1.05-1.20)	933.09	947	1.01 (0.95-1.08)	68.59	78	1.18 (0.90-1.42)
45	Sale of motor vehicles and parts	113.44	133	1.17 (0.98-1.42)	109.19	118	1.08 (0.89-1.29)	11.39	15	1.32 (0.74-2.17)
46	Wholesale trade on own account or on a fee or contract basis	1,441.20	1,372	0.95 (0.90-1.00)	1,181.02	1,142	0.97 (0.91-1.02)	255.17	230	0.90 (0.79-1.03)
47	Retail trade, except motor vehicles and motorcycles	1,439.08	1,351	0.94 (0.89-0.99)	1,098.58	1,058	0.96 (0.91-1.02)	300.16	293	0.98 (0.87-1.09)
49	Land transport and transport via pipelines	428.41	553	1.29 (1.17-1.42)	468.00	526	1.12 (1.03-1.22)	19.91	27	1.36 (0.89-1.97)
50	Water transport	125.27	158	1.26 (1.06-1.51)	141.47	155	1.10 (0.93-1.28)	4.27	3	0.70 (0.15-2.06)
51	Air transport	15.75	17	1.08 (0.66-1.90)	16.07	16	1.00 (0.57-1.62)	1.41	1	0.71 (0.02-3.98)
52	Warehousing and support activities for transportation	1,651.72	2,065	1.25 (1.19-1.31)	1,845.13	2,004	1.09 (1.04-1.13)	62.24	61	098 (0.75-1.26)
55	Accommodation	161.30	138	0.86 (0.73-1.00)	92.57	91	0.98 (0.79-1.21)	41.6	4	1.13 (0.83-1.50)
56	Food and beverage service activities	614.44	385	0.63 (0.58-0.68)	199.93	179	0.90 (0.77-1.04)	225.21	206	0.91 (0.79-1.05)
59	Motion picture, video and television program production, sound recording and music publishing activities	20.86	17	0.81 (0.53-1.32)	19.17	14	0.73 (0.40-1.23)	3.04	3	0.99 (0.20-2.88)
60	Broadcasting activities	15.66	20	1.28 (0.78-2.25)	9.56	9	0.94 (0.43-1.79)	5.25	11	2.10 (1.05-3.75)
61	Postal activities and telecommunications	205.13	221	1.08 (0.94-1.24)	209.52	203	0.97 (0.84-1.11)	17.18	18	1.05 (0.62-1.66)
62	Computer programming, consultancy and related activities	125.20	102	0.81 (0.68-0.98)	111.90	83	0.74 (0.59-0.92)	19.57	19	0.97 (0.58-1.52)
63	Information service activities	186.89	177	0.95 (0.82-1.10)	164.11	151	0.92 (0.78-1.08)	30.49	26	0.85 (0.56-1.25)
64	Financial service activities, except insurance and pension funding	767.09	817	1.07 (0.99-1.14)	692.90	672	0.97 (0.90-1.05)	135.39	145	1.07 (0.90-1.26)

65	Insurance and pension funding	96.92	104	1.07 (0.88-1.32)	87.79	73	0.83 (0.65-1.05)	17.76	31	1.75 (1.19-2.48)
66	Activities auxiliary to financial service and insurance activities	44.29	43	0.97 (0.72-1.33)	37.22	30	0.81 (0.54-1.15)	8.81	13	1.48 (0.79-2.52)
68	Real estate activities	1,864.00	1,837	0.99 (0.94-1.03)	1,579.60	1,588	1.01 (0.96-1.06)	259.97	249	0.96 (0.84-1.08)
70	Research and development	17.28	17	0.98 (0.62-1.68)	15.57	15	0.96 (0.54-1.59)	2.81	2	0.71 (0.09-2.57)
71	Professional services	410.43	340	0.83 (0.75-0.91)	332.62	270	0.81 (0.72-0.91)	89.69	70	0.78 (0.61-0.99)
72	Architectural, engineering and other scientific technical services	359.33	363	1.01 (0.91-1.12)	366.25	334	0.91 (0.82-1.02)	29.53	29	0.98 (0.66-1.41)
73	Other professional, scientific and technical services	43.65	37	0.85 (0.63-1.17)	35.85	31	0.86 (0.59-1.23)	8.15	6	0.74 (0.27-1.60)
74	Business facilities management and landscape services	562.40	491	0.87 (0.80-0.95)	359.50	393	1.09 (0.99-1.21)	116.54	98	0.84 (0.68-1.02)
75	Business support services	1,368.34	1,284	0.94 (0.89-0.99)	977.07	1,018	1.04 (0.98-1.11)	288.74	266	0.92 (0.81-1.04)
76	Rental and leasing activities; except real estate	63.22	65	1.03 (0.80-1.34)	59.78	59	0.99 (0.75-1.27)	7.35	6	0.82 (0.30-1.78)
84	Public administration and defence; compulsory social security	251.97	271	1.08 (0.95-1.21)	210.56	229	1.09 (0.95-1.24)	43.12	42	0.97 (0.70-1.32)
85	Education	863.67	649	0.75 (0.70-0.80)	269.94	249	0.92 (0.81-1.04)	408.60	400	0.98 (0.89-1.08)
86	Human health activities	943.98	741	0.78 (0.74-0.84)	480.39	393	0.82 (0.74-0.90)	340.55	348	1.02 (0.92-1.14)
87	Social work activities	652.30	455	0.70 (0.65-0.75)	194.28	185	0.95 (0.82-1.10)	270.98	270	1.00 (0.88-1.12)
90	Creative, arts and recreation related services	27.24	25	0.92 (0.63-1.39)	19.53	17	0.87 (0.51-1.39)	6.45	8	1.24 (0.54-2.44)
91	Sports activities and amusement activities	389.16	376	0.97 (0.87-1.07)	295.45	297	1.01 (0.89-1.13)	71.74	79	1.10 (0.87-1.37)
94	Membership organizations	732.76	653	0.89 (0.83-0.96)	565.04	513	0.91 (0.83-0.99)	136.95	140	1.02 (0.86-1.21)
95	Maintenance and repair services of personal and household goods	166.44	190	1.14 (0.98-1.32)	163.75	175	1.07 (0.92-1.24)	16.01	15	0.94 (0.52-1.55)
96	Other personal services activities	627.27	610	0.97 (0.90-1.05)	444.31	474	1.07 (0.97-1.17)	136.38	136	1.00 (0.84-1.18)
98	Undifferentiated goods-and services-producing activities of private households for own use	207.30	208	1.00 (0.87-1.15)	179.40	176	0.98 (0.84-1.14)	28.60	32	1.12 (0.77-1.58)
99	Activities of extraterritorial organizations and bodies	68.05	71	1.04 (0.81-1.32)	62.29	61	0.98 (0.75-1.26)	8.06	10	1.24 (0.60-2.28)
	Non-confirmed	4,935.20	5,079	1.03 (1.00-1.06)	4,200.30	4,184	1.00 (0.97-1.03)	850.09	895	1.05 (0.98-1.12)

Supplementary Table S5. Age-standardized incidence ratio (SIR) and 95% confidence interval (CI) of malignant neoplasm of colon according to divisions of the Korean Standard Industrial Classification (KSIC).

KSIC		Total workers			Male workers			Female workers		
Divisions	Description	Expected cases	Observed cases	SIR (95% CI)	Expected cases	Observed cases	SIR (95% CI)	Expected cases	Observed cases	SIR (95% CI)
01	Agriculture	27.06	32	1.18 (0.81-1.79)	21.36	24	1.12 (0.72-1.67)	5.51	8	1.45 (0.63-2.86)
02	Forestry	6.48	8	1.23 (0.58-3.21)	6.30	8	1.27 (0.55-2.50)	0.63	0	0.00 (0.00-0.00)
03	Fishing and aquaculture	17.71	23	1.30 (0.82-2.20)	14.63	18	1.23 (0.73-1.94)	3.50	5	1.43 (0.46-3.33)
05	Mining of coal, crude petroleum and natural gas	25.42	33	1.30 (0.88-2.00)	24.71	29	1.17 (0.79-1.69)	2.23	4	1.80 (0.49-4.60)
06	Mining of metal ores	2.23	3	1.35 (0.40-9.46)	2.14	3	1.40 (0.29-4.10)	0.21	0	0.00 (0.00-0.00)
07	Mining of non-metallic minerals, except fuel	8.94	13	1.45 (0.76-3.19)	8.91	11	1.23 (0.62-2.21)	0.66	2	3.02 (0.37-10.91)
08	Mining support service activities	2.49	3	1.21 (0.38-7.30)	2.29	2	0.87 (0.11-3.16)	0.31	1	3.26 (0.08-18.15)
10	Manufacture of food products	186.76	172	0.92 (0.80-1.07)	99.69	97	0.97 (0.79-1.19)	69.48	75	1.08 (0.85-1.35)
11	Manufacture of beverages	7.89	5	0.63 (0.32-1.48)	6.54	4	0.61 (0.17-1.56)	1.37	1	0.73 (0.02-4.06)
12	Manufacture of tobacco products	0.57	2	3.48 (0.42-12.59)	0.49	1	2.06 (0.05-11.49)	0.09	1	10.75 (0.27-59.91)
13	Manufacture of textiles, except apparel	169.00	149	0.88 (0.76-1.03)	114.93	95	0.83 (0.67-1.01)	47.81	54	1.13 (0.85-1.47)
14	Manufacture of wearing apparel, clothing accessories and fur articles	45.56	28	0.61 (0.46-0.84)	22.59	14	0.62 (0.34-1.04)	18.55	14	0.75 (0.41-1.27)
15	Manufacture of leather, luggage and footwear	31.35	35	1.12 (0.79-1.64)	20.81	27	1.30 (0.85-1.89)	9.09	8	0.88 (0.38-1.73)
16	Manufacture of wood and of products of wood and cork; except furniture	48.02	40	0.83 (0.63-1.13)	42.24	34	0.80 (0.56-1.12)	7.13	6	0.84 (0.31-1.83)
17	Manufacture of pulp, paper and paper products	51.06	65	1.27 (0.97-1.71)	44.79	56	1.25 (0.94-1.62)	7.49	9	1.20 (0.55-2.28)
18	Printing and reproduction of recorded media	106.98	92	0.86 (0.71-1.05)	84.53	74	0.88 (0.69-1.10)	23.48	18	0.77 (0.45-1.21)
19	Manufacture of coke, briquettes and refined petroleum products	12.37	15	1.21 (0.70-2.32)	12.65	14	1.11 (0.61-1.86)	0.53	1	1.89 (0.05-10.51)
20	Manufacture of chemicals and chemical products; except pharmaceuticals and medicinal chemicals	174.06	160	0.92 (0.79-1.07)	151.62	126	0.83 (0.69-0.99)	26.32	34	1.29 (0.89-1.81)
21	Manufacture of pharmaceuticals, medicinal chemical and botanical products	12.28	11	0.90 (0.52-1.72)	9.38	8	0.85 (0.37-1.68)	2.8	3	1.07 (0.22-3.13)
22	Manufacture of rubber and plastics products	66.72	65	0.97 (0.77-1.26)	53.67	47	0.88 (0.64-1.16)	13.29	18	1.35 (0.80-2.14)
23	Manufacture of other non-metallic mineral products	77.11	71	0.92 (0.74-1.17)	70.50	60	0.85 (0.65-1.10)	9.49	11	1.16 (0.58-2.07)
24	Manufacture of basic metals	177.28	189	1.07 (0.92-1.24)	171.59	170	0.99 (0.85-1.15)	15.48	19	1.23 (0.74-1.92)
25	Manufacture of fabricated metal products, except machinery and furniture	162.95	162	0.99 (0.85-1.17)	146.18	144	0.99 (0.83-1.16)	22.13	18	0.81 (0.48-1.29)
26	Manufacture of electronic components, computer; visual, sounding and communication equipment	214.16	211	0.99 (0.86-1.13)	153.87	153	0.99 (0.84-1.16)	57.39	58	1.01 (0.77-1.31)

27	Manufacture of medical, precision and optical instruments, watches and clocks	23.07	24	1.04 (0.69-1.64)	17.44	15	0.86 (0.48-1.42)	5.52	9	1.63 (0.75-3.09)
28	Manufacture of electrical equipment	98.63	91	0.92 (0.76-1.14)	83.43	77	0.92 (0.73-1.15)	17.11	14	0.82 (0.45-1.37)
29	Manufacture of other machinery and equipment	220.40	222	1.01 (0.88-1.15)	201.12	200	0.99 (0.86-1.14)	27.64	22	0.80 (0.50-1.21)
30	Manufacture of motor vehicles, trailers and semitrailers	322.18	305	0.95 (0.85-1.06)	280.54	256	0.91 (0.80-1.03)	47.65	49	1.03 (0.76-1.36)
31	Manufacture of other transport equipment	177.61	181	1.02 (0.88-1.19)	117.01	161	0.91 (0.77-1.06)	12.26	20	1.63 (1.00-2.52)
32	Manufacture of furniture	33.75	22	0.65 (0.47-0.94)	28.06	20	0.71 (0.44-1.10)	6.01	2	0.33 (0.04-1.20)
33	Other manufacturing	1,137.55	1,056	0.93 (0.88-0.98)	941.57	866	0.92 (0.86-0.98)	206.63	190	0.92 (0.79-1.06)
35	Electricity, gas, steam and air conditioning supply	114.53	111	0.97 (0.81-1.17)	116.43	107	0.92 (0.75-1.11)	6.48	4	0.62 (0.17-1.58)
36	Water supply	7.57	7	0.92 (0.46-2.20)	7.57	6	0.79 (0.29-1.73)	0.51	1	1.95 (0.05-10.86)
37	Sewage, wastewater, human and animal waste treatment services	6.69	4	0.60 (0.29-1.53)	5.44	4	0.74 (0.20-1.88)	1.20	0	0.00 (0.00-0.00)
38	Waste collection, treatment and disposal activities; materials recovery	36.54	47	1.29 (0.93-1.83)	32.86	43	1.31 (0.95-1.76)	4.86	4	0.82 (0.22-2.11)
41	General construction	635.30	683	1.08 (0.99-1.16)	617.40	620	1.00 (0.93-1.09)	54.10	63	1.16 (0.89-1.49
42	Specialized construction activities	386.62	411	1.06 (0.96-1.18)	372.66	367	0.98 (0.89-1.09)	35.90	44	1.23 (0.89-1.65)
45	Sale of motor vehicles and parts	46.83	54	1.15 (0.87-1.57)	42.65	47	1.10 (0.81-1.47)	5.89	7	1.19 (0.48-2.45)
46	Wholesale trade on own account or on a fee or contract basis	605.67	553	0.91 (0.84-0.99)	471.04	428	0.91 (0.82-1.00)	134.33	125	0.93 (0.77-1.11)
47	Retail trade, except motor vehicles and motorcycles	601.60	577	0.96 (0.89-1.04)	438.80	424	0.97 (0.88-1.06)	155.04	153	0.99 (0.84-1.16)
49	Land transport and transport via pipelines	186.71	231	1.24 (1.07-1.44)	190.99	225	1.18 (1.03-1.34)	10.48	6	0.57 (0.21-1.25)
50	Water transport	56.74	69	1.22 (0.94-1.61)	60.44	68	1.13 (0.87-1.43)	1.96	1	0.51 (0.01-2.84)
51	Air transport	6.51	12	1.84 (0.87-4.78)	6.27	10	1.60 (0.77-2.93)	0.63	2	3.18 (0.39-11.50)
52	Warehousing and support activities for transportation	729.71	908	1.24 (1.16-1.34)	762.44	880	1.15 (1.08-1.23)	32.08	28	0.87 (0.58-1.26)
55	Accommodation	69.76	67	0.96 (0.76-1.23)	37.10	45	1.21 (0.88-1.62)	25.48	22	0.86 (0.54-1.31)
56	Food and beverage service activities	266.06	178	0.67 (0.59-0.76)	80.25	66	0.81 (0.67-0.97)	138.43	112	0.82 (0.64-1.05)
59	Motion picture, video and television program production, sound recording and music publishing activities	8.37	8	0.96 (0.41-1.88)	7.32	6	0.82 (0.30-1.78)	1.31	2	1.53 (0.18-5.51)
60	Broadcasting activities	6.33	7	1.11 (0.52-2.91)	3.78	3	0.79 (0.16-2.32)	2.37	4	1.69 (0.46-4.32)
61	Postal activities and telecommunications	82.61	70	0.85 (0.68-1.06)	78.70	59	0.75 (0.57-0.97)	8.24	11	1.34 (0.67-2.39)
62	Computer programming, consultancy and related activities	48.01	43	0.90 (0.68-1.21)	41.43	36	0.87 (0.61-1.20)	7.76	7	0.90 (0.36-1.86)
63	Information service activities	72.28	69	0.95 (0.76-1.22)	61.26	61	1.00 (0.76-1.28)	12.54	8	0.64 (0.28-1.26)
64	Financial service activities, except insurance and pension funding	307.74	253	0.82 (0.72-0.93)	263.94	200	0.76 (0.66-0.87)	56.62	53	0.94 (0.70-1.22)

65	Insurance and pension funding	38.07	41	1.08 (0.78-1.52)	32.71	32	0.98 (0.67-1.38)	7.07	9	1.27 (0.58-2.42)
66	Activities auxiliary to financial service and insurance activities	17.79	10	0.56 (0.27-1.03)	14.15	9	0.64 (0.29-1.21)	3.90	1	0.26 (0.01-1.43)
68	Real estate activities	860.30	909	1.06 (0.99-1.13)	689.70	749	1.09 (1.01-1.17)	162.85	160	0.98 (0.84-1.15)
70	Research and development	7.03	9	1.28 (0.62-3.17)	6.05	8	1.32 (0.57-2.61)	1.22	1	0.82 (0.02-4.57)
71	Professional services	172.13	187	1.09 (0.94-1.27)	134.99	143	1.06 (0.89-1.25)	40.68	44	1.08 (0.79-1.45)
72	Architectural, engineering and other scientific technical services	155.32	183	1.18 (1.01-1.36)	150.40	167	1.11 (0.95-1.29)	14.50	16	1.10 (0.63-1.79)
73	Other professional, scientific and technical services	17.98	24	1.33 (0.84-2.25)	14.11	19	1.35 (0.81-2.10)	3.92	5	1.27 (0.41-2.97)
74	Business facilities management and landscape services	265.20	258	0.97 (0.86-1.10)	160.79	163	1.01 (0.86-1.18)	78.90	95	1.20 (0.97-1.47)
75	Business support services	614.39	636	1.04 (0.96-1.12)	421.43	441	1.05 (0.95-1.15)	167.71	195	1.16 (1.01-1.34)
76	Rental and leasing activities; except real estate	26.78	24	0.90 (0.61-1.36)	24.10	22	0.91 (0.57-1.38)	3.69	2	0.54 (0.07-1.96)
84	Public administration and defence; compulsory social security	109.74	123	1.12 (0.93-1.36)	87.20	101	1.16 (0.94-1.41)	23.49	22	0.94 (0.59-1.42)
85	Education	353.94	309	0.87 (0.79-0.97)	111.47	98	0.88 (0.71-1.07)	203.41	211	1.04 (0.90-1.19)
86	Human health activities	396.60	343	0.86 (0.78-0.96)	193.21	197	1.02 (0.88-1.17)	170.63	146	0.86 (0.72-1.01)
87	Social work activities	279.76	241	0.86 (0.77-0.97)	79.55	91	1.14 (0.92-1.40)	153.72	150	0.98 (0.83-1.15)
90	Creative, arts and recreation related services	11.50	9	0.78 (0.44-1.54)	7.89	6	0.76 (0.28-1.65)	3.26	3	0.92 (0.19-2.69)
91	Sports activities and amusement activities	175.41	190	1.08 (0.93-1.26)	126.84	136	1.07 (0.90-1.27)	42.72	54	1.26 (0.95-1.65)
94	Membership organizations	323.62	300	0.93 (0.83-1.04)	235.81	228	0.97 (0.85-1.10)	79.12	72	0.91 (0.71-1.15)
95	Maintenance and repair services of personal and household goods	66.14	62	0.91 (0.70-1.17)	63.57	58	0.91 (0.69-1.18)	7.61	4	0.53 (0.14-1.35)
96	Other personal services activities	276.21	265	0.96 (0.85-1.08)	187.34	188	1.00 (0.87-1.16)	77.97	77	0.99 (0.78-1.23)
98	Undifferentiated goods-and services-producing activities of private households for own use	97.97	102	1.04 (0.85-1.26)	81.41	85	1.04 (0.83-1.29)	17.56	17	0.97 (0.56-1.55)
99	Activities of extraterritorial organizations and bodies	30.52	44	1.44 (1.05-1.94)	26.19	38	1.45 (1.03-1.99)	4.90	6	1.23 (0.45-2.67)
	Non-confirmed	2,090.80	2,200	1.05 (1.01-1.10)	1.682.50	1748	1.04 (0.99-1.09)	440.79	452	1.03 (0.93-1.13)

Supplementary Table S6. Age-standardized incidence ratio (SIR) and 95% confidence interval (CI) of malignant neoplasm of rectosigmoid junction, rectum, anus and anal canal according to divisions of the Korean Standard Industrial Classification (KSIC).

KSIC		Total workers			Male workers			Female workers		
Divisions	Description	Expected cases	Observed cases	SIR (95% CI)	Expected cases	Observed cases	SIR (95% CI)	Expected cases	Observed cases	SIR (95% CI)
01	Agriculture	22.41	17	0.76 (0.50-1.20)	18.47	15	0.81 (0.45-1.34)	3.82	2	0.52 (0.06-1.89)
02	Forestry	5.23	8	1.53 (0.67-4.56)	5.23	8	1.53 (0.66-3.02)	0.45	0	0.00 (0.00-0.00)
03	Fishing and aquaculture	14.81	11	0.74 (0.45-1.33)	12.55	9	0.72 (0.33-1.36)	2.55	2	0.79 (0.10-2.84)
05	Mining of coal, crude petroleum and natural gas	21.00	26	1.24 (0.81-2.00)	21.30	23	1.08 (0.68-1.62)	1.57	3	1.92 (0.40-5.60)
06	Mining of metal ores	1.86	1	0.54 (0.14-5.03)	1.86	1	0.54 (0.01-2.99)	0.15	0	0.00 (0.00-0.00)
07	Mining of non-metallic minerals, except fuel	7.26	9	1.24 (0.61-3.02)	7.51	8	1.07 (0.46-2.10)	0.46	1	2.16 (0.05-12.01)
08	Mining support service activities	2.03	1	0.49 (0.14-4.00)	1.93	1	0.52 (0.01-2.88)	0.21	0	0.00 (0.00-0.00)
10	Manufacture of food products	158.09	146	0.92 (0.79-1.09)	89.60	92	1.03 (0.83-1.26)	48.46	54	1.11 (0.84-1.45)
11	Manufacture of beverages	6.79	4	0.59 (0.28-1.49)	5.95	4	0.67 (0.18-1.72)	0.97	0	0.00 (0.00-0.00)
12	Manufacture of tobacco products	0.51	0	0.00 (0.00-0.00)	0.45	0	0.00 (0.00-0.00)	0.07	0	0.00 (0.00-0.00)
13	Manufacture of textiles, except apparel	143.18	119	0.83 (0.71-0.99)	101.86	88	0.86 (0.69-1.06)	33.84	31	0.92 (0.62-1.30)
14	Manufacture of wearing apparel, clothing accessories and fur articles	38.85	39	1.00 (0.73-1.41)	20.31	23	1.13 (0.72-1.70)	13.20	16	1.21 (0.69-1.97)
15	Manufacture of leather, luggage and footwear	26.32	28	1.06 (0.73-1.62)	18.30	17	0.93 (0.54-1.49)	6.36	11	1.73 (0.86-3.10)
16	Manufacture of wood and of products of wood and cork; except furniture	39.89	67	1.68 (1.23-2.35)	36.52	63	1.73 (1.33-2.21)	5.00	4	0.80 (0.22-2.05)
17	Manufacture of pulp, paper and paper products	43.25	50	1.16 (0.86-1.60)	39.81	45	1.13 (0.82-1.51)	5.28	5	0.95 (0.31-2.21)
18	Printing and reproduction of recorded media	93.16	90	0.97 (0.79-1.20)	96.63	74	0.97 (0.76-1.21)	17.55	16	0.91 (0.52-1.48)
19	Manufacture of coke, briquettes and refined petroleum products	10.98	13	1.18 (0.66-2.37)	11.84	12	1.01 (0.52-1.77)	0.40	1	2.48 (0.06-13.83)
20	Manufacture of chemicals and chemical products; except pharmaceuticals and medicinal chemicals	150.56	153	1.02 (0.87-1.20)	137.88	130	0.94 (0.79-1.12)	19.05	23	1.21 (0.77-1.81)
21	Manufacture of pharmaceuticals, medicinal chemical and botanical products	10.78	10	0.93 (0.52-1.87)	8.68	8	0.92 (0.40-1.82)	2.06	2	0.97 (0.12-3.50)
22	Manufacture of rubber and plastics products	56.91	60	1.05 (0.81-1.39)	47.88	51	1.07 (0.79-1.40)	9.46	9	0.95 (0.43-1.81)
23	Manufacture of other non-metallic mineral products	64.96	72	1.11 (0.87-1.44)	62.14	65	1.05 (0.81-1.33)	6.69	7	1.05 (0.42-2.16)
24	Manufacture of basic metals	150.74	175	1.16 (0.99-1.37)	152.85	166	1.09 (0.93-1.26)	11.04	9	0.81 (0.37-1.55)
25	Manufacture of fabricated metal products, except machinery and furniture	139.65	125	0.90 (0.76-1.06)	131.20	111	0.85 (0.70-1.02)	15.86	14	0.88 (0.48-1.48)
26	Manufacture of electronic components, computer; visual, sounding and communication equipment	193.97	184	0.95 (0.82-1.10)	147.30	150	1.02 (0.86-1.19)	43.25	34	0.79 (0.54-1.10)

27	Manufacture of medical, precision and optical instruments, watches and clocks	20.30	23	1.13 (0.74-1.85)	16.08	18	1.12 (0.66-1.77)	4.06	5	1.23 (0.40-2.88)
28	Manufacture of electrical equipment	85.52	102	1.19 (0.97-1.49)	75.73	85	1.12 (0.90-1.39)	12.45	17	1.37 (0.80-2.19)
29	Manufacture of other machinery and equipment	190.17	177	0.93 (0.80-1.08)	181.85	165	0.91 (0.77-1.06)	20.03	12	0.60 (0.31-1.05)
30	Manufacture of motor vehicles, trailers and semitrailers	284.52	282	0.99 (0.88-1.11)	262.58	246	0.94 (0.82-1.06)	34.12	36	1.06 (0.74-1.46)
31	Manufacture of other transport equipment	151.54	135	0.89 (0.76-1.05)	158.11	127	0.80 (0.67-0.96)	8.82	8	0.91 (0.39-1.79)
32	Manufacture of furniture	28.78	20	0.70 (0.48-1.04)	25.12	17	0.68 (0.39-1.08)	4.24	3	0.71 (0.15-2.07)
33	Other manufacturing	979.52	1,027	1.05 (0.98-1.12)	851.39	868	1.02 (0.95-1.09)	148.63	159	1.07 (0.91-1.25)
35	Electricity, gas, steam and air conditioning supply	98.15	98	1.00 (0.82-1.23)	104.55	93	0.89 (0.72-1.09)	4.82	5	1.04 (0.34-2.42)
36	Water supply	6.53	3	0.46 (0.22-1.19)	6.84	3	0.44 (0.09-1.28)	0.38	0	0.00 (0.00-0.00)
37	Sewage, wastewater, human and animal waste treatment services	5.36	2	0.37 (0.16-1.10)	4.56	2	0.44 (0.05-1.58)	0.80	0	0.00 (0.00-0.00)
38	Waste collection, treatment and disposal activities; materials recovery	29.81	36	1.21 (0.85-1.79)	27.89	33	1.18 (0.81-1.66)	3.34	3	0.90 (0.19-2.62)
41	General construction	534.50	579	1.08 (1.00-1.18)	542.90	537	0.99 (0.91-1.08)	38.86	42	1.08 (0.78-1.46)
42	Specialized construction activities	327.44	341	1.04 (0.93-1.16)	329.54	312	0.95 (0.84-1.06)	26.11	29	1.11 (0.74-1.60)
45	Sale of motor vehicles and parts	40.73	39	0.96 (0.71-1.34)	38.93	35	0.90 (0.63-1.25)	4.31	4	0.93 (0.25-2.38)
46	Wholesale trade on own account or on a fee or contract basis	518.56	494	0.95 (0.87-1.04)	420.23	402	0.96 (0.87-1.05)	97.86	92	0.94 (0.76-1.15)
47	Retail trade, except motor vehicles and motorcycles	517.12	507	0.98 (0.90-1.07)	392.09	393	1.00 (0.91-1.11)	113.58	114	1.00 (0.83-1.21)
49	Land transport and transport via pipelines	154.88	173	1.12 (0.95-1.32)	165.02	164	0.99 (0.85-1.16)	7.60	9	1.18 (0.54-2.25)
50	Water transport	45.49	46	1.01 (0.76-1.38)	50.08	46	0.92 (0.67-1.23)	1.51	0	0.00 (0.00-0.00)
51	Air transport	5.67	12	2.12 (0.95-5.98)	5.69	11	1.93 (0.97-3.46)	0.50	1	2.02 (0.05-11.23)
52	Warehousing and support activities for transportation	598.06	788	1.32 (1.22-1.43)	649.61	767	1.18 (1.10-1.27)	23.50	21	0.89 (0.55-1.37)
55	Accommodation	58.42	42	0.72 (0.56-0.95)	33.07	30	0.91 (0.61-1.29)	17.59	12	0.68 (0.35-1.19)
56	Food and beverage service activities	222.09	154	0.69 (0.61-0.79)	71.50	60	0.84 (0.64-1.08)	95.57	94	0.98 (0.79-1.20)
59	Motion picture, video and television program production, sound recording and music publishing activities	7.18	4	0.53 (0.15-1.37)	6.86	4	0.58 (0.16-1.49)	1.03	0	0.00 (0.00-0.00)
60	Broadcasting activities	5.66	6	1.06 (0.48-3.00)	3.47	1	0.29 (0.01-1.60	1.83	5	2.73 (0.89-6.37)
61	Postal activities and telecommunications	73.40	65	0.89 (0.70-1.13)	73.57	59	0.80 (0.61-1.03)	6.18	6	0.97 (0.36-2.11)
62	Computer programming, consultancy and related activities	44.90	64	1.43 (1.10-1.82)	40.94	55	1.34 (1.01-1.75)	6.36	9	1.42 (0.65-2.69)
63	Information service activities	66.99	74	1.10 (0.87-1.43)	59.95	62	1.03 (0.79-1.33)	10.08	12	1.19 (0.61-2.08)
64	Financial service activities, except insurance and pension funding	274.97	240	0.87 (0.77-0.99)	244.58	196	0.80 (0.69-0.92)	45.09	44	0.98 (0.71-1.31)

65	Insurance and pension funding	34.62	33	0.95 (0.68-1.37)	30.87	29	0.94 (0.63-1.35)	5.70	4	0.70 (0.19-1.80)
66	Activities auxiliary to financial service and insurance activities	15.85	14	0.88 (0.48-1.48)	13.17	13	0.99 (0.53-1.69)	3.02	1	0.33 (0.01-1.84)
68	Real estate activities	674.50	714	1.06 (0.98-1.14)	558.00	608	1.09 (1.00-1.18)	109.39	106	0.97 (0.79-1.17)
70	Research and development	6.21	5	0.80 (0.37-2.15)	5.55	5	0.90 (0.29-2.10)	0.96	0	0.00 (0.00-0.00)
71	Professional services	147.57	137	0.93 (0.79-1.10)	117.91	114	0.97 (0.80-1.16)	31.31	23	0.73 (0.47-1.10)
72	Architectural, engineering and other scientific technical services	129.68	131	1.01 (0.84-1.20)	130.20	121	0.93 (0.77-1.11)	10.77	10	0.93 (0.45-1.71)
73	Other professional, scientific and technical services	15.69	22	1.40 (0.86-2.47)	12.88	13	1.01 (0.54-1.73)	2.95	9	3.05 (1.40-5.79)
74	Business facilities management and landscape services	203.80	187	0.92 (0.80-1.06)	127.12	130	1.02 (0.85-1.21)	51.58	57	1.11 (0.84-1.43)
75	Business support services	495.38	532	1.07 (0.98-1.18)	347.67	403	1.16 (1.05-1.28)	117.08	129	1.10 (0.92-1.31)
76	Rental and leasing activities; except real estate	22.82	20	0.88 (0.58-1.38)	21.39	17	0.79 (0.46-1.27)	2.72	3	1.10 (0.23-3.22)
84	Public administration and defence; compulsory social security	91.02	92	1.01 (0.82-1.26)	74.43	72	0.97 (0.76-1.22)	16.83	20	1.19 (0.73-1.84)
85	Education	309.14	240	0.78 (0.69-0.87)	95.94	94	0.98 (0.79-1.20)	151.29	146	0.97 (0.81-1.13)
86	Human health activities	339.67	333	0.98 (0.88-1.09)	169.53	198	1.17 (1.01-1.34)	126.00	135	1.07 (0.90-1.27)
87	Social work activities	235.56	169	0.72 (0.63-0.82)	69.08	72	1.04 (0.82-1.31)	108.79	97	0.89 (0.72-1.09)
90	Creative, arts and recreation related services	9.81	13	1.33 (0.72-2.79)	6.99	12	1.72 (0.89-3.00)	2.41	1	0.42 (0.01-2.32)
91	Sports activities and amusement activities	140.76	159	1.13 (0.96-1.34)	104.92	119	1.13 (0.94-1.36)	29.49	40	1.36 (0.97-1.85)
94	Membership organizations	264.63	246	0.93 (0.82-1.05)	199.85	192	0.96 (0.83-1.11)	55.24	54	0.98 (0.73-1.28
95	Maintenance and repair services of personal and household goods	59.73	50	0.84 (0.62-1.10)	58.42	47	0.80 (0.59-1.07)	5.78	3	0.52 (0.11-1.52)
96	Other personal services activities	226.52	224	0.99 (0.87-1.13)	157.90	169	1.07 (0.91-1.24)	54.85	55	1.00 (0.76-1.31)
98	Undifferentiated goods-and services-producing activities of private households for own use	75.06	91	1.21 (0.98-1.49)	63.25	78	1.23 (0.97-1.54)	11.91	13	1.09 (0.58-1.87)
99	Activities of extraterritorial organizations and bodies	24.64	19	0.77 (0.46-1.20)	21.95	18	0.30 (0.01-1.66)	3.36	1	0.82 (0.49-1.30)
	Non-confirmed	1,780.70	1,711	0.96 (0.92-1.01)	1,484.1	1,393	0.94 (0.89-0.99)	322.58	318	0.99 (0.88-1.10)

Supplementary Table S7. Age-standardized incidence ratio (SIR) and 95% confidence interval (CI) of malignant neoplasm of liver and intrahepatic bile ducts according to divisions of the Korean Standard Industrial Classification (KSIC).

KSIC		Total workers			Male workers			Female workers		
Divisions	Description	Expected cases	Observed cases	SIR (95% CI)	Expected cases	Observed cases	SIR (95% CI)	Expected cases	Observed cases	SIR (95% CI)
01	Agriculture	34.42	31	0.90 (0.65-1.30)	31.53	29	0.92 (0.62-1.32)	2.66	2	0.75 (0.09-2.72)
02	Forestry	7.99	22	2.75 (1.40-6.38)	8.81	21	2.38 (1.48-3.64)	0.28	1	3.53 (0.09-19.69)
03	Fishing and aquaculture	23.11	37	1.60 (1.07-2.52)	21.70	34	1.57 (1.09-2.19)	1.55	3	1.94 (0.40-5.67)
05	Mining of coal, crude petroleum and natural gas	33.19	51	1.54 (1.10-2.23)	37.78	50	1.32 (0.98-1.74)	1.06	1	0.94 (0.02-5.25)
06	Mining of metal ores	2.90	5	1.72 (0.58-8.66)	3.26	5	1.53 (0.50-3.58)	0.10	0	0.00 (0.00-0.00)
07	Mining of non-metallic minerals, except fuel	11.28	13	1.15 (0.65-2.28)	12.96	13	1.00 (0.53-1.72)	0.32	0	0.00 (0.00-0.00)
08	Mining support service activities	3.17	6	1.89 (0.66-8.63)	3.37	6	1.78 (0.65-3.88)	0.15	0	0.00 (0.00-0.00)
10	Manufacture of food products	245.73	178	0.72 (0.64-0.82)	154.48	145	0.94 (0.79-1.10)	32.99	33	1.00 (0.69-1.40)
11	Manufacture of beverages	10.37	6	0.58 (0.32-1.19)	10.37	6	0.58 (0.21-1.26)	0.64	0	0.00 (0.00-0.00)
12	Manufacture of tobacco products	0.74	1	1.35 (0.20-160.20)	0.75	1	1.34 (0.03-7.45)	0.04	0	0.00 (0.00-0.00)
13	Manufacture of textiles, except apparel	223.35	213	0.95 (0.84-1.09)	178.92	188	1.05 (0.91-1.21)	`22.17	25	1.13 (0.73-1.66)
14	Manufacture of wearing apparel, clothing accessories and fur articles	60.04	39	0.65 (0.50-0.85)	35.52	33	0.93 (0.64-1.30)	8.58	6	0.70 (0.26-1.52)
15	Manufacture of leather, luggage and footwear	41.34	40	0.97 (0.71-1.35)	32.30	32	0.99 (0.68-1.40)	4.27	8	1.87 (0.81-3.68)
16	Manufacture of wood and of products of wood and cork; except furniture	61.79	77	1.25 (0.97-1.63)	63.13	73	1.16 (0.91-1.45)	3.36	4	1.19 (0.32-3.05)
17	Manufacture of pulp, paper and paper products	67.24	81	1.20 (0.95-1.55)	69.96	78	1.11 (0.88-1.39)	3.51	3	0.85 (0.18-2.50)
18	Printing and reproduction of recorded media	140.67	144	1.02 (0.87-1.22)	133.60	133	1.00 (0.83-1.18)	10.02	11	1.10 (0.55-1.96)
19	Manufacture of coke, briquettes and refined petroleum products	17.23	17	0.99 (0.62-1.69)	21.59	17	0.79 (0.46-1.26)	0.23	0	0.00 (0.00-0.00)
20	Manufacture of chemicals and chemical products; except pharmaceuticals and medicinal chemicals	229.07	201	0.88 (0.77-1.00)	239.27	189	0.79 (0.68-0.91)	11.98	12	1.00 (0.52-1.75)
21	Manufacture of pharmaceuticals, medicinal chemical and botanical products	15.96	10	0.63 (0.39-1.10)	14.75	9	0.61 (0.28-1.16)	1.24	1	0.81 (0.02-4.49)
22	Manufacture of rubber and plastics products	87.76	98	1.12 (0.91-1.39)	82.94	92	1.11 (0.89-1.36)	6.12	6	0.98 (0.36-2.13)
23	Manufacture of other non-metallic mineral products	100.29	101	1.01 (0.83-1.24)	108.02	96	0.89 (0.72-1.09)	4.44	5	1.13 (0.37-2.63)
24	Manufacture of basic metals	235.02	284	1.21 (1.06-1.38)	269.70	275	1.02 (0.90-1.15)	7.12	9	1.26 (0.58-2.40)
25	Manufacture of fabricated metal products, except machinery and furniture	215.31	256	1.19 (1.04-1.37)	228.71	246	1.08 (0.95-1.22)	10.08	10	0.99 (0.48-1.82)
26	Manufacture of electronic components, computer; visual, sounding and communication equipment	282.63	209	0.74 (0.66-0.83)	245.22	189	0.77 (0.66-0.89)	24.41	20	0.82 (0.50-1.27)

27	Manufacture of medical, precision and optical instruments, watches and clocks	30.54	25	0.82 (0.58-1.21)	27.45	23	0.84 (0.53-1.26)	2.41	2	0.83 (0.10-3.00)
28	Manufacture of electrical equipment	130.49	131	1.00 (0.85-1.20)	131.03	122	0.93 (0.77-1.11)	7.62	9	1.18 (0.54-2.24)
29	Manufacture of other machinery and equipment	291.70	346	1.19 (1.06-1.33)	317.20	333	1.05 (0.94-1.17)	12.39	13	1.05 (0.56-1.79)
30	Manufacture of motor vehicles, trailers and semitrailers	441.85	378	0.86 (0.78-0.94)	469.27	358	0.77 (0.69-0.85)	21.61	20	0.93 (0.57-1.43)
31	Manufacture of other transport equipment	235.76	282	1.20 (1.05-1.36)	278.14	277	1.00 (0.88-1.12)	5.55	5	0.90 (0.29-2.10)
32	Manufacture of furniture	44.61	48	1.08 (0.80-1.48)	43.97	46	1.05 (0.77-1.40)	2.79	2	0.72 (0.09-2.59)
33	Other manufacturing	1,489.90	1,498	1.01 (0.96-1.06)	1,468.58	1,413	0.96 (0.91-1.01)	94.24	85	0.90 (0.72-1.12)
35	Electricity, gas, steam and air conditioning supply	154.12	188	1.22 (1.04-1.44)	186.75	185	0.99 (0.85-1.14)	2.72	3	1.10 (0.23-3.22)
36	Water supply	10.41	11	1.06 (0.58-2.17)	12.48	11	0.88 (0.44-1.58)	0.22	0	0.00 (0.00-0.00)
37	Sewage, wastewater, human and animal waste treatment services	8.18	13	1.59 (0.81-3.64)	7.73	12	1.55 (0.80-2.71)	0.62	1	1.61 (0.04-8.94)
38	Waste collection, treatment and disposal activities; materials recovery	45.87	62	1.35 (1.01-1.85)	47.68	58	1.22 (0.92-1.57)	2.38	4	1.68 (0.46-4.31)
41	General construction	817.70	1087	1.33 (1.24-1.43)	941.90	1,052	1.12 (1.05-1.19)	24.83	35	1.41 (0.98-1.96)
42	Specialized construction activities	504.80	755	1.50 (1.37-1.64)	576.28	740	1.28 (1.19-1.38)	15.95	15	0.94 (0.53-1.55)
45	Sale of motor vehicles and parts	61.22	83	1.36 (1.06-1.77)	68.89	78	1.17 (0.92-1.46)	2.59	5	1.93 (0.63-4.51)
46	Wholesale trade on own account or on a fee or contract basis	782.93	792	1.01 (0.94-1.09)	719.45	732	1.02 (0.95-1.09)	60.14	60	1.00 (0.76-1.28)
47	Retail trade, except motor vehicles and motorcycles	772.52	766	0.99 (0.92-1.07)	662.16	688	1.04 (0.96-1.12)	68.84	78	1.13 (0.90-1.41)
49	Land transport and transport via pipelines	242.23	307	1.27 (1.12-1.44)	289.65	301	1.04 (0.93-1.16)	4.69	6	1.28 (0.47-2.78)
50	Water transport	68.85	95	1.38 (1.09-1.77)	83.96	95	1.13 (0.92-1.38)	0.846	0	0.00 (0.00-0.00)
51	Air transport	8.67	6	0.69 (0.36-1.54)	10.07	6	0.60 (0.22-1.30)	0.26	0	0.00 (0.00-0.00)
52	Warehousing and support activities for transportation	939.19	1,290	1.37 (1.29-1.47)	1,139.64	1,284	1.13 (1.07-1.19)	14.28	6	0.42 (0.15-0.91)
55	Accommodation	89.13	63	0.71 (0.57-0.88)	55.98	42	0.75 (0.54-1.01)	12.37	21	1.70 (1.05-2.59)
56	Food and beverage service activities	347.28	184	0.53 (0.48-0.59)	121.08	113	0.93 (0.77-1.12)	66.76	71	1.06 (0.83-1.34)
59	Motion picture, video and television program production, sound recording and music publishing activities	10.78	8	0.74 (0.32-1.46)	11.68	8	0.68 (0.30-1.35)	0.53	0	0.00 (0.00-0.00)
60	Broadcasting activities	7.85	4	0.51 (0.26-1.19)	5.63	4	0.71 (0.19-1.82)	0.96	0	0.00 (0.00-0.00)
61	Postal activities and telecommunications	116.76	135	1.16 (0.96-1.40)	136.12	131	0.96 (0.80-1.14)	3.33	4	1.20 (0.33-3.08)
62	Computer programming, consultancy and related activities	59.90	57	0.95 (0.72-1.23)	65.81	55	0.84 (0.63-1.09)	2.95	2	0.68 (0.08-2.45)
63	Information service activities	90.35	91	1.01 (0.82-1.25)	96.61	87	0.90 (0.72-1.11)	4.89	4	0.82 (0.22-2.10)
64	Financial service activities, except insurance and pension funding	414.52	443	1.07 (0.97-1.17)	439.67	417	0.95 (0.86-1.04)	21.39	26	1.22 (0.79-1.78)

65	Insurance and pension funding	52.70	29	0.55 (0.42-0.74)	56.86	27	0.47 (0.31-0.69)	2.62	2	0.76 (0.09-2.76)
66	Activities auxiliary to financial service and insurance activities	23.29	14	0.60 (0.33-1.01)	23.11	14	0.61 (0.33-1.02)	1.59	0	0.00 (0.00-0.00)
68	Real estate activities	1,002.10	1,041	1.04 (0.98-1.11)	904.00	940	1.04 (0.97-1.11)	84.53	101	1.19 (0.97-1.45)
70	Research and development	9.05	7	0.77 (0.41-1.69)	9.51	7	0.74 (0.30-1.52)	0.45	0	0.00 (0.00-0.00)
71	Professional services	216.39	198	0.92 (0.80-1.05)	199.59	181	0.91 (0.78-1.05)	16.70	17	1.02 (0.59-1.63)
72	Architectural, engineering and other scientific technical services	193.61	215	1.11 (0.97-1.27)	219.48	214	0.98 (0.85-1.11)	6.37	1	0.16 (0.00-0.87)
73	Other professional, scientific and technical services	22.60	24	1.06 (0.71-1.68)	21.41	23	1.07 (0.68-1.61)	1.69	1	0.59 (0.01-3.29)
74	Business facilities management and landscape services	303.90	279	0.92 (0.82-1.03)	201.90	230	1.14 (1.00-1.30)	42.59	49	1.15 (0.85-1.52)
75	Business support services	736.84	683	0.93 (0.86-1.00)	563.52	583	1.03 (0.95-1.12)	81.81	100	1.22 (0.99-1.49)
76	Rental and leasing activities; except real estate	33.78	46	1.36 (0.97-1.97)	36.05	44	1.22 (0.89-1.64)	1.62	2	1.23 (0.15-4.45)
84	Public administration and defence; compulsory social security	140.78	155	1.10 (0.93-1.31)	128.56	148	1.15 (0.97-1.35)	10.68	7	0.66 (0.26-1.35)
85	Education	465.69	190	0.41 (0.37-0.45)	160.86	138	0.86 (0.72-1.01)	87.20	52	0.60 (0.45-0.78)
86	Human health activities	502.21	308	0.61 (0.56-0.67)	291.93	229	0.78 (0.69-0.89)	75.85	79	1.04 (0.82-1.30)
87	Social work activities	361.10	178	0.49 (0.44-0.55)	116.45	108	0.93 (0.76-1.12)	71.67	70	0.98 (0.76-1.23)
90	Creative, arts and recreation related services	14.07	8	0.57 (0.34-1.04)	11.44	8	0.70 (0.30-1.38)	1.47	0	0.00 (0.00-0.00)
91	Sports activities and amusement activities	208.88	208	1.00 (0.87-1.15)	170.40	183	1.07 (0.92-1.24)	21.28	25	1.17 (0.76-1.73)
94	Membership organizations	400.98	378	0.94 (0.85-1.04)	337.29	338	1.00 (0.90-1.11)	38.28	40	1.04 (0.75-1.42)
95	Maintenance and repair services of personal and household goods	88.76	128	1.44 (1.20-1.71)	101.14	124	1.24 (1.03-1.48)	3.24	4	1.24 (0.34-3.17)
96	Other personal services activities	341.21	332	0.97 (0.88-1.09)	26.16	299	1.14 (1.02-1.28)	37.06	33	0.89 (0.61-1.25)
98	Undifferentiated goods-and services-producing activities of private households for own use	110.60	134	1.21 (1.02-1.43)	99.39	119	1.20 (0.99-1.43)	8.93	15	1.68 (0.94-2.77)
99	Activities of extraterritorial organizations and bodies	38.12	45	1.18 (0.86-1.58)	37.60	44	1.17 (0.85-1.57)	2.40	1	0.42 (0.01-2.32)
	Non-confirmed	2,780.00	2,547	0.92 (0.88-0.95)	2,639.00	2,377	0.90 (0.86-0.94)	192.44	170	0.88 (0.76-1.03)

Supplementary Table S8. Age-standardized incidence ratio (SIR) and 95% confidence interval (CI) of malignant neoplasm of pancreas according to divisions of the Korean Standard Industrial Classification (KSIC).

KSIC		Total workers			Male workers			Female workers		
Divisions	Description	Expected cases	Observed cases	SIR (95% CI)	Expected cases	Observed cases	SIR (95% CI)	Expected cases	Observed cases	SIR (95% CI)
01	Agriculture	8.01	6	0.75 (0.38-1.73)	6.51	4	0.61 (0.17-1.57)	1.46	2	1.37 (0.17-4.94)
02	Forestry	1.95	2	1.03 (0.28-8.83)	1.94	1	0.52 (0.01-2.87)	0.15	1	6.49 (0.16-36.18)
03	Fishing and aquaculture	5.22	7	1.34 (0.58-4.01)	4.48	7	1.56 (0.63-3.22)	0.83	0	0.00 (0.00-0.00)
05	Mining of coal, crude petroleum and natural gas	7.56	11	1.46 (0.73-2.60)	7.57	10	1.32 (0.63-2.43)	0.58	1	1.72 (0.04-9.59)
06	Mining of metal ores	0.66	1	1.52 (0.04-8.44)	0.65	1	1.53 (0.04-8.53)	0.06	0	0.00 (0.00-0.00)
07	Mining of non-metallic minerals, except fuel	2.69	3	1.12 (0.36-6.14)	2.75	3	1.09 (0.23-3.19)	0.17	0	0.00 (0.00-0.00)
08	Mining support service activities	0.75	0	0.00 (0.00-0.00)	0.70	0	0.00 (0.00-0.00)	0.08	0	0.00 (0.00-0.00)
10	Manufacture of food products	54.41	57	1.05 (0.80-1.39)	29.98	33	1.10 (0.76-1.55)	18.07	24	1.33 (0.85-1.98)
11	Manufacture of beverages	2.27	4	1.76 (0.52-12.06)	1.97	4	2.02 (0.55-5.21)	0.35	0	0.00 (0.00-0.00)
12	Manufacture of tobacco products	0.16	0	0.00 (0.00-0.00)	0.14	0	0.00 (0.00-0.00)	0.02	0	0.00 (0.00-0.00)
13	Manufacture of textiles, except apparel	49.31	35	0.71 (0.54-0.96)	34.88	21	0.60 (0.37-0.92)	12.08	14	1.16 (0.63-1.94)
14	Manufacture of wearing apparel, clothing accessories and fur articles	13.18	17	1.29 (0.76-2.41)	6.81	10	1.47 (0.70-2.70)	4.67	7	1.50 (0.60-3.09)
15	Manufacture of leather, luggage and footwear	9.20	11	1.20 (0.63-2.59)	6.32	9	1.42 (0.65-2.70)	2.35	2	0.85 (0.10-3.08)
16	Manufacture of wood and of products of wood and cork; except furniture	14.17	14	0.99 (0.59-1.80)	12.87	12	0.93 (0.48-1.63)	1.84	2	1.09 (0.13-3.93)
17	Manufacture of pulp, paper and paper products	14.91	13	0.87 (0.53-1.56)	13.59	11	0.81 (0.40-1.45)	1.92	2	1.04 (0.13-3.76)
18	Printing and reproduction of recorded media	30.45	29	0.95 (0.67-1.41)	25.45	24	0.94 (0.60-1.40)	5.35	5	0.93 (0.30-2.18)
19	Manufacture of coke, briquettes and refined petroleum products	3.52	4	1.14 (0.42-4.68)	3.82	4	1.05 (0.29-2.68)	0.12	0	0.00 (0.00-0.00)
20	Manufacture of chemicals and chemical products; except pharmaceuticals and medicinal chemicals	49.81	49	0.98 (0.75-1.33)	45.44	42	0.92 (0.67-1.25)	6.45	7	1.08 (0.44-2.24)
21	Manufacture of pharmaceuticals, medicinal chemical and botanical products	3.45	4	1.16 (0.42-4.87)	2.78	4	1.44 (0.39-3.69)	0.66	0	0.00 (0.00-0.00)
22	Manufacture of rubber and plastics products	19.35	25	1.29 (0.83-2.14)	16.17	21	1.30 (0.80-1.99)	3.33	4	1.20 (0.33-3.08)
23	Manufacture of other non-metallic mineral products	22.55	29	1.29 (0.85-2.04)	21.38	26	1.22 (0.79-1.78)	2.43	3	1.24 (0.26-3.62)
24	Manufacture of basic metals	51.59	57	1.10 (0.84-1.48)	51.95	53	1.02 (0.76-1.33)	3.67	4	1.03 (0.28-2.65)
25	Manufacture of fabricated metal products, except machinery and furniture	47.05	45	0.96 (0.72-1.30)	43.99	40	0.91 (0.65-1.24)	5.46	5	0.92 (0.30-2.14)

26	Manufacture of electronic components, computer; visual, sounding and communication equipment	58.59	42	0.72 (0.56-0.94)	44.66	30	0.67 (0.45-0.96)	12.83	12	0.94 (0.48-1.63)
27	Manufacture of medical, precision and optical instruments, watches and clocks	6.51	7	1.08 (0.51-2.79)	5.18	4	0.77 (0.21-1.98)	1.29	3	2.33 (0.48-6.81)
28	Manufacture of electrical equipment	28.18	30	1.06 (0.74-1.60)	24.96	26	1.04 (0.68-1.53)	4.09	4	0.98 (0.27-2.50)
29	Manufacture of other machinery and equipment	63.23	64	1.01 (0.79-1.32)	60.34	56	0.93 (0.70-1.21)	6.68	8	1.20 (0.52-2.36)
30	Manufacture of motor vehicles, trailers and semitrailers	91.35	83	0.91 (0.74-1.13)	83.71	73	0.87 (0.68-1.10)	11.70	10	0.85 (0.41-1.57)
31	Manufacture of other transport equipment	51.48	59	1.15 (0.87-1.54)	53.36	53	0.99 (0.74-1.30)	3.01	6	2.00 (0.73-4.34)
32	Manufacture of furniture	9.79	6	0.61 (0.33-1.29)	8.46	5	0.59 (0.19-1.38)	1.52	1	0.66 (0.02-3.66)
33	Other manufacturing	326.47	347	1.06 (0.95-1.19)	282.41	303	1.07 (0.96-1.20)	50.95	44	0.86 (0.63-1.16)
35	Electricity, gas, steam and air conditioning supply	33.26	39	1.17 (0.84-1.70)	35.35	39	1.10 (0.78-1.51)	1.47	0	0.00 (0.00-0.00)
36	Water supply	2.19	5	2.29 (0.66-16.50)	2.29	4	1.75 (0.48-4.48)	0.12	1	8.40 (0.21-46.82)
37	Sewage, wastewater, human and animal waste treatment services	2.03	3	1.48 (0.41-11.97)	1.68	2	1.19 (0.14-4.30)	0.35	1	2.89 (0.07-16.10)
38	Waste collection, treatment and disposal activities; materials recovery	10.94	13	1.19 (0.66-2.39)	10.10	13	1.29 (0.69-2.20)	1.31	0	0.00 (0.00-0.00
41	General construction	186.12	197	1.06 (0.92-1.23)	187.96	181	0.96 (0.83-1.11)	13.51	16	1.18 (0.68-1.92)
42	Specialized construction activities	112.83	121	1.07 (0.89-1.30)	113.28	110	0.97 (0.80-1.17)	8.64	11	1.27 (0.64-2.28)
45	Sale of motor vehicles and parts	13.34	15	1.12 (0.66-2.09)	12.76	14	1.10 (0.60-1.84)	1.39	1	0.72 (0.02-4.00)
46	Wholesale trade on own account or on a fee or contract basis	174.55	166	0.95 (0.82-1.11)	142.06	131	0.92 (0.77-1.09)	32.39	35	1.08 (0.75-1.50)
47	Retail trade, except motor vehicles and motorcycles	172.53	167	0.97 (0.83-1.13)	131.90	131	0.99 (0.83-1.18)	36.92	36	0.98 (0.68-1.35)
49	Land transport and transport via pipelines	55.40	56	1.01 (0.78-1.34)	58.54	53	0.91 (0.68-1.18)	2.54	3	1.18 (0.24-3.46)
50	Water transport	17.11	24	1.40 (0.88-2.40)	18.64	24	1.29 (0.82-1.92)	0.44	0	0.00 (0.00-0.00)
51	Air transport	1.85	1	0.54 (0.14-5.06)	1.89	1	0.53 (0.01-2.96)	0.13	0	0.00 (0.00-0.00)
52	Warehousing and support activities for transportation	218.35	265	1.21 (1.06-1.39)	234.68	259	1.10 (0.97-1.25)	7.70	6	0.78 (0.29-1.70)
55	Accommodation	20.41	16	0.78 (0.51-1.28)	11.16	10	0.90 (0.43-1.65)	6.81	6	0.88 (0.32-1.92)
56	Food and beverage service activities	78.36	60	0.77 (0.61-0.97)	24.09	22	0.91 (0.57-1.38)	36.69	38	1.04 (0.73-1.42)
59	Motion picture, video and television program production, sound recording and music publishing activities	2.31	3	1.30 (0.39-8.68)	2.17	3	1.38 (0.29-4.05)	0.28	0	0.00 (0.00-0.00)
60	Broadcasting activities	1.73	2	1.16 (0.29-12.23)	1.11	2	1.80 (0.22-6.51)	0.51	0	0.00 (0.00-0.00)
61	Postal activities and telecommunications	23.38	12	0.51 (0.34-0.81)	23.62	10	0.42 (0.20-0.78)	1.79	2	1.12 (0.14-4.03)
62	Computer programming, consultancy and related activities	12.54	14	1.12 (0.61-1.87)	11.84	12	1.01 (0.52-1.77)	1.49	2	1.34 (0.16-4.85)

63	Information service activities	19.10	21	1.10 (0.70-1.82)	17.60	20	1.14 (0.69-1.75)	2.50	1	0.40 (0.01-2.23)
64	Financial service activities, except insurance and pension funding	85.85	95	1.11 (0.90-1.35)	79.33	85	1.07 (0.86-1.32)	11.16	10	0.90 (0.43-1.65)
65	Insurance and pension funding	10.51	20	1.90 (1.05-3.89)	9.79	17	1.74 (1.01-2.78)	1.34	3	2.23 (0.46-6.53)
66	Activities auxiliary to financial service and insurance activities	4.96	4	0.81 (0.22-2.07)	4.24	4	0.94 (0.26-2.42)	0.84	0	0.00 (0.00-0.00)
68	Real estate activities	262.62	245	0.93 (0.82-1.06)	213.78	204	0.95 (0.83-1.09)	46.71	41	0.88 (0.63-1.19)
70	Research and development	1.97	3	1.52 (0.42-12.91)	1.81	2	1.10 (0.13-3.99)	0.26	1	3.92 (0.10-21.85)
71	Professional services	49.28	45	0.91 (0.69-1.23)	41.03	39	0.95 (0.68-1.30)	8.79	6	0.68 (0.25-1.49)
72	Architectural, engineering and other scientific technical services	45.47	70	1.54 (1.20-1.94)	45.75	67	1.46 (1.13-1.86)	3.41	3	0.88 (0.18-2.57)
73	Other professional, scientific and technical services	5.06	4	0.79 (0.34-2.41)	4.20	3	0.71 (0.15-2.09)	0.9	1	1.11 (0.03-6.19)
74	Business facilities management and landscape services	81.92	78	0.95 (0.77-1.20)	50.04	54	1.08 (0.81-1.41)	23.69	24	1.01 (0.65-1.51)
75	Business support services	183.84	191	1.04 (0.90-1.21)	129.44	144	1.11 (0.94-1.31)	44.62	47	1.05 (0.77-1.40)
76	Rental and leasing activities; except real estate	7.71	11	1.43 (0.72-3.37)	7.25	10	1.38 (0.66-2.54)	0.87	1	1.15 (0.03-6.38)
84	Public administration and defence; compulsory social security	32.49	23	0.71 (0.50-1.03)	26.77	21	0.78 (0.49-1.20)	5.80	2	0.35 (0.04-1.25)
85	Education	100.54	95	0.94 (0.78-1.16)	33.92	36	1.06 (0.74-1.47)	46.47	59	1.27 (0.97-1.64)
86	Human health activities	113.59	103	0.91 (0.75-1.10)	54.81	66	1.12 (0.87-1.43)	40.52	37	0.91 (0.64-1.26)
87	Social work activities	81.68	60	0.73 (0.59-0.92)	24.19	25	1.03 (0.67-1.53)	39.05	35	0.90 (0.62-1.25)
90	Creative, arts and recreation related services	3.28	3	0.92 (0.33-4.05)	2.36	3	1.27 (0.26-3.71)	0.78	0	0.00 (0.00-0.00)
91	Sports activities and amusement activities	52.66	48	0.91 (0.70-1.22)	38.98	38	0.97 (0.69-1.34)	11.66	10	0.86 (0.41-1.58)
94	Membership organizations	96.37	85	0.88 (0.72-1.09)	72.33	67	0.93 (0.72-1.18)	20.94	18	0.86 (0.51-1.36)
95	Maintenance and repair services of personal and household goods	19.26	18	0.93 (0.55-1.48)	19.00	18	0.95 (0.56-1.50)	1.71	0	0.00 (0.00-0.00
96	Other personal services activities	81.90	74	0.90 (0.73-1.14)	57.30	57	0.99 (0.75-1.29)	20.20	17	0.84 (0.49-1.35)
98	Undifferentiated goods-and services-producing activities of private households for own use	30.28	27	0.89 (0.59-1.30)	25.45	22	0.86 (0.54-1.31)	4.93	5	1.02 (0.33-2.37)
99	Activities of extraterritorial organizations and bodies	9.21	4	0.43 (0.12-1.11)	8.10	3	0.37 (0.08-1.08)	1.32	1	0.76 (0.02-4.21)
	Non-confirmed	608.44	608	1.00 (0.92-1.08)	512.24	498	0.97 (0.89-1.06)	103.73	110	1.06 (0.87-1.28)

Supplementary Table S9. Age-standardized incidence ratio (SIR) and 95% confidence interval (CI) of other malignant neoplasm of digestive organs according to divisions of the Korean Standard Industrial Classification (KSIC).

KSIC		Total workers			Male workers			Female workers		
Divisions	Description	Expected cases	Observed cases	SIR (95% CI)	Expected cases	Observed cases	SIR (95% CI)	Expected cases	Observed cases	SIR (95% CI)
01	Agriculture	8.33	10	1.20 (0.62-2.72)	6.53	6	0.92 (0.34-2.00)	1.77	4	2.26 (0.62-5.78)
02	Forestry	2.04	3	1.47 (0.41-11.76)	1.97	3	1.52 (0.31-4.45)	0.19	0	0.00 (0.00-0.00)
03	Fishing and aquaculture	5.40	7	1.30 (0.57-3.78)	4.49	6	1.34 (0.49-2.91)	1.00	1	1.00 (0.03-5.55)
05	Mining of coal, crude petroleum and natural gas	7.84	11	1.40 (0.71-3.28)	7.56	10	1.32 (0.63-2.43)	0.70	1	1.42 (0.04-7.93)
06	Mining of metal ores	0.68	0	0.00 (0.00-0.00)	0.65	0	0.00 (0.00-0.00)	0.07	0	0.00 (0.00-0.00)
07	Mining of non-metallic minerals, except fuel	2.81	1	0.36 (0.12-1.86)	2.77	1	0.36 (0.01-2.01)	0.21	0	0.00 (0.00-0.00)
08	Mining support service activities	0.78	0	0.00 (0.00-0.00)	0.71	0	0.00 (0.00-0.00	0.10	0	0.00 (0.00-0.00
10	Manufacture of food products	56.18	66	1.17 (0.91-1.55)	29.72	46	1.55 (1.13-2.06)	21.78	20	0.92 (0.56-1.42)
11	Manufacture of beverages	2.34	2	0.85 (0.26-5.61)	1.94	2	1.03 (0.13-3.73)	0.42	0	0.00 (0.00-0.00)
12	Manufacture of tobacco products	0.17	0	0.00 (0.00-0.00)	0.14	0	0.00 (0.00-0.00)	0.03	0	0.00 (0.00-0.00)
13	Manufacture of textiles, except apparel	50.85	49	0.96 (0.73-1.29)	34.55	34	0.98 (0.68-1.38)	14.60	15	1.03 (0.58-1.69)
14	Manufacture of wearing apparel, clothing accessories and fur articles	13.60	6	0.44 (0.26-0.82)	6.73	2	0.30 (0.04-1.07)	5.63	4	0.71 (0.19-1.82)
15	Manufacture of leather, luggage and footwear	9.50	9	0.95 (0.51-2.02)	6.29	5	0.79 (0.26-1.85)	2.83	4	1.41 (0.38-3.62)
16	Manufacture of wood and of products of wood and cork; except furniture	14.72	11	0.75 (0.45-1.34)	12.88	8	0.62 (0.27-1.22)	2.22	3	1.35 (0.28-3.95)
17	Manufacture of pulp, paper and paper products	15.39	21	1.36 (0.83-2.42)	13.47	17	1.26 (0.74-2.02)	2.32	4	1.72 (0.47-4.42)
18	Printing and reproduction of recorded media	31.33	16	0.51 (0.36-0.75)	25.09	14	0.56 (0.31-0.94)	6.46	2	0.31 (0.04-1.12)
19	Manufacture of coke, briquettes and refined petroleum products	3.56	5	1.40 (0.52-5.72)	3.66	5	1.36 (0.44-3.18)	0.14	0	0.00 (0.00-0.00)
20	Manufacture of chemicals and chemical products; except pharmaceuticals and medicinal chemicals	51.30	47	0.92 (0.70-1.23)	44.85	41	0.91 (0.66-1.24)	7.78	6	0.77 (0.28-1.68)
21	Manufacture of pharmaceuticals, medicinal chemical and botanical products	3.55	2	0.56 (0.21-2.30)	2.74	1	0.36 (0.01-2.03)	0.80	1	1.25 (0.03-6.96)
22	Manufacture of rubber and plastics products	19.96	15	0.75 (0.49-1.23)	16.07	13	0.81 (0.43-1.38)	4.02	2	0.50 (0.06-1.80)
23	Manufacture of other non-metallic mineral products	23.34	21	0.90 (0.60-1.41)	21.27	17	0.80 (0.47-1.28)	2.94	4	1.36 (0.37-3.49)
24	Manufacture of basic metals	53.14	61	1.15 (0.88-1.53)	51.33	56	1.09 (0.82-1.42)	4.68	5	1.07 (0.35-2.50)
25	Manufacture of fabricated metal products, except machinery and furniture	48.47	68	1.40 (1.06-1.90)	43.56	63	1.45 (1.11-1.85)	6.59	5	0.76 (0.25-1.77)
26	Manufacture of electronic components, computer; visual, sounding and communication equipment	59.92	55	0.92 (0.71-1.20)	43.96	38	0.86 (0.61-1.19)	15.51	17	1.10 (0.64-1.75)

27	Manufacture of medical, precision and optical instruments, watches and clocks	6.68	7	1.05 (0.42-2.16)	5.11	4	0.78 (0.21-2.00)	1.56	3	1.92 (0.40-5.62)
28	Manufacture of electrical equipment	28.99	21	0.72 (0.50-1.08)	24.69	17	0.69 (0.40-1.10)	4.95	4	0.81 (0.22-2.07)
29	Manufacture of other machinery and equipment	65.06	67	1.03 (0.81-1.33)	59.62	55	0.92 (0.69-1.20)	8.07	12	1.49 (0.77-2.60)
30	Manufacture of motor vehicles, trailers and semitrailers	93.05	109	1.17 (0.96-1.45)	81.27	91	1.12 (0.90-1.37)	14.11	18	1.28 (0.76-2.02)
31	Manufacture of other transport equipment	53.05	55	1.04 (0.79-1.38)	52.84	51	0.97 (0.72-1.27)	3.64	4	1.10 (0.30-2.82)
32	Manufacture of furniture	10.09	12	1.19 (0.65-2.47)	8.38	12	1.43 (0.74-2.50)	1.84	0	0.00 (0.00-0.00)
33	Other manufacturing	336.66	323	0.96 (0.86-1.07)	279.63	268	0.96 (0.85-1.08)	61.40	55	0.90 (0.67-1.17)
35	Electricity, gas, steam and air conditioning supply	34.17	35	1.02 (0.73-1.48)	34.79	34	0.98 (0.68-1.37)	1.78	1	0.56 (0.01-3.14)
36	Water supply	2.24	1	0.45 (0.13-3.12)	2.24	1	0.45 (0.01-2.48)	0.14	0	0.00 (0.00-0.00)
37	Sewage, wastewater, human and animal waste treatment services	2.13	2	0.94 (0.27-7.05)	1.70	1	0.59 (0.01-3.28)	0.42	1	2.37 (0.06-13.20
38	Waste collection, treatment and disposal activities; materials recovery	11.42	13	1.14 (0.64-2.25)	10.16	11	1.08 (0.54-1.94)	1.59	2	1.26 (0.15-4.55)
41	General construction	192.92	194	1.01 (0.87-1.16)	186.95	182	0.97 (0.84-1.13)	16.40	12	0.73 (0.38-1.28)
42	Specialized construction activities	116.60	124	1.06 (0.89-1.29)	112.41	109	0.97 (0.80-1.17)	10.47	15	1.43 (0.80-2.36)
45	Sale of motor vehicles and parts	13.75	14	1.02 (0.60-1.87)	12.61	11	0.87 (0.44-.56)	1.69	3	1.78 (0.37-5.20)
46	Wholesale trade on own account or on a fee or contract basis	180.47	171	0.95 (0.82-1.10)	141.27	134	0.95 (0.79-1.12)	39.15	37	0.95 (0.67-1.30)
47	Retail trade, except motor vehicles and motorcycles	178.45	167	0.94 (0.81-1.09)	131.51	130	0.99 (0.83-1.17)	44.75	37	0.83 (0.58-1.14)
49	Land transport and transport via pipelines	57.46	73	1.27 (0.98-1.68)	58.40	70	1.20 (0.93-1.51)	3.08	3	0.97 (0.20-2.85)
50	Water transport	17.98	31	1.72 (1.09-2.91)	18.93	31	1.64 (1.11-2.32)	0.53	0	0.00 (0.00-0.00)
51	Air transport	1.90	3	1.58 (0.42-14.14)	1.86	3	1.62 (0.33-4.72)	0.16	0	0.00 (0.00-0.00)
52	Warehousing and support activities for transportation	227.08	252	1.11 (0.97-1.27)	235.07	245	1.04 (0.92-1.18)	9.29	7	0.75 (0.30-1.55)
55	Accommodation	21.20	23	1.08 (0.71-1.75)	11.13	17	1.53 (0.89-2.44)	8.22	6	0.73 (0.27-1.59)
56	Food and beverage service activities	81.17	56	0.69 (0.56-0.87)	24.03	14	0.58 (0.32-0.98)	44.37	42	0.95 (0.68-1.28)
59	Motion picture, video and television program production, sound recording and music publishing activities	2.38	0	0.00 (0.00-0.00)	2.13	0	0.00 (0.00-0.00)	0.33	0	0.00 (0.00-0.00)
60	Broadcasting activities	1.79	2	1.11 (0.29-11.02)	1.11	2	1.80 (0.22-6.51)	0.61	0	0.00 (0.00-0.00)
61	Postal activities and telecommunications	23.73	22	0.93 (0.62-1.45)	22.93	22	0.96 (0.60-1.45)	2.17	0	0.00 (0.00-0.00)
62	Computer programming, consultancy and related activities	12.94	14	1.08 (0.59-1.82)	11.65	14	1.20 (0.66-2.02)	1.76	0	0.00 (0.00-0.00)
63	Information service activities	19.70	12	0.61 (0.39-1.00)	17.34	12	0.69 (0.36-1.21)	2.98	0	0.00 (0.00-0.00)
64	Financial service activities, except insurance and pension funding	87.69	105	1.20 (0.98-1.45)	77.23	84	1.09 (0.87-1.35)	13.39	21	1.57 (0.97-2.40)

65	Insurance and pension funding	10.65	10	0.94 (0.52-1.91)	9.44	8	0.85 (0.37-1.67)	1.63	2	1.22 (0.15-4.42)
66	Activities auxiliary to financial service and insurance activities	5.09	4	0.79 (0.21-2.01)	4.13	4	0.97 (0.26-2.48)	1.01	0	0.00 (0.00-0.00)
68	Real estate activities	277.96	272	0.98 (0.87-1.10)	219.23	207	0.94 (0.82-1.08)	57.06	65	1.14 (0.88-1.45)
70	Research and development	2.03	0	0.00 (0.00-0.00)	1.79	0	0.00 (0.00-0.00)	0.31	0	0.00 (0.00-0.00)
71	Professional services	51.16	46	0.90 (0.68-1.21)	41.11	37	0.90 (0.63-1.24)	10.59	9	0.85 (0.39-1.61)
72	Architectural, engineering and other scientific technical services	47.42	46	0.97 (0.71-1.29)	45.99	39	0.85 (0.60-1.16)	4.12	7	1.70 (0.68-3.50)
73	Other professional, scientific and technical services	5.25	5	0.95 (0.42-2.84)	4.18	4	0.96 (0.26-2.45)	1.08	1	0.92 (0.02-5.14)
74	Business facilities management and landscape services	87.01	93	1.07 (0.87-1.33)	51.70	64	1.24 (0.95-1.58)	29.00	29	1.00 (0.67-1.44)
75	Business support services	193.35	198	1.02 (0.89-1.19)	132.31	139	1.05 (0.88-1.24)	54.17	59	1.09 (0.83-1.40)
76	Rental and leasing activities; except real estate	8.00	9	1.12 (0.57-2.60)	7.24	9	1.24 (0.57-2.36)	1.05	0	0.00 (0.00-0.00)
84	Public administration and defence; compulsory social security	33.74	27	0.80 (0.57-1.16)	26.84	20	0.75 (0.46-1.15)	7.04	7	0.99 (0.40-2.05)
85	Education	103.25	86	0.83 (0.69-1.02)	34.14	29	0.85 (0.57-1.22)	56.43	57	1.01 (0.77-1.31)
86	Human health activities	117.69	105	0.89 (0.74-1.08)	58.58	55	0.94 (0.71-1.22)	49.08	50	1.02 (0.76-1.34)
87	Social work activities	84.63	74	0.87 (0.71-1.10)	24.29	15	0.62 (0.35-1.02)	47.29	59	1.25 (0.95-1.61)
90	Creative, arts and recreation related services	3.41	4	1.17 (0.43-4.97)	2.37	2	0.84 (0.10-3.05)	0.94	2	2.12 (0.26-7.65)
91	Sports activities and amusement activities	55.46	78	1.41 (1.08-1.86)	39.77	58	1.46 (1.11-1.89)	14.17	20	1.41 (0.86-2.18)
94	Membership organizations	100.78	104	1.03 (0.85-1.27)	72.99	72	0.99 (0.77-1.24)	25.46	32	1.26 (0.86-1.77)
95	Maintenance and repair services of personal and household goods	19.83	31	1.56 (1.06-2.22)	18.73	26	1.39 (0.91-2.03)	2.05	5	2.44 (0.79-5.69)
96	Other personal services activities	85.63	82	0.96 (0.76-1.19)	58.10	63	1.08 (0.83-1.39)	24.52	19	0.77 (0.47-1.21)
98	Undifferentiated goods-and services-producing activities of private households for own use	32.21	40	1.24 (0.89-1.69)	26.38	33	1.25 (0.86-1.76)	6.01	7	1.16 (0.47-2.40)
99	Activities of extraterritorial organizations and bodies	9.63	14	1.45 (0.79-2.44)	1.61	2	1.47 (0.76-2.56)	8.18	12	1.25 (0.15-4.50)
	Non-confirmed	626.88	605	0.97 (0.89-1.05)	507.63	496	0.98 (0.89-1.07)	125.84	109	0.87 (0.71-1.04)
